# The Ca^2+^‐CAMTA6‐PP2C49‐HKT1;1 signaling axis modulates ion homeostasis during Arabidopsis seed germination under salt stress

**DOI:** 10.1111/tpj.71033

**Published:** 2026-07-09

**Authors:** Yvonne Kiere, Ancy E. J. Chandran, Guy Sobol, Omer Cremer, Shaked Azoulay‐Portal, Yonatan Wexler, Doron Shkolnik

**Affiliations:** ^1^ Robert H. Smith Institute of Plant Sciences and Genetics in Agriculture, Faculty of Agriculture, Food and Environment Hebrew University of Jerusalem Rehovot 7610001 Israel; ^2^ Present address: Department of Bacteriology University of Wisconsin–Madison Madison Wisconsin 53706 USA

**Keywords:** *Arabidopsis thaliana*, Ca^2+^, Na^+^, K^+^, CAMTA6, PP2C49, HKT1;1

## Abstract

Soil salinization severely constrains seedling establishment by disrupting cellular Na^+^/K^+^ homeostasis. Calcium (Ca^2+^) signaling contributes to the re‐establishment of ion balance during early growth, yet the mechanisms linking Ca^2+^ perception to ion transport regulation remain unclear. Here, we identify a Ca^2+^‐responsive regulatory module in *Arabidopsis thaliana* comprising CALMODULIN‐BINDING TRANSCRIPTION ACTIVATOR 6 (CAMTA6), the TYPE 2C PROTEIN PHOSPHATASE PP2C49, and the HIGH‐AFFINITY K^+^ TRANSPORTER HKT1;1, and define their coordinated roles during germination under salt stress. Spatial promoter analyses revealed that NaCl induces *CAMTA6* expression at cotyledon margins, while CaCl_2_ stimulates *HKT1;1* transcription in the radicle, consistent with CAMTA6‐mediated repression of *HKT1;1*. In *camta6* mutants, *PP2C49* expression expanded beyond its normal radicle‐restricted domain, indicating CAMTA6‐dependent spatial control. Promoter activation assays *in planta* demonstrated CAMTA6‐dependent transactivation of the *HKT1;1* and *PP2C49* promoters. Treatment with the PP2C inhibitor sanguinarine enhanced germination under salinity in the wild type, but not in *hkt1* nor in the salt‐tolerant *camta6* and *pp2c49* mutants. Sanguinarine restricted *CAMTA6* promoter activity to cotyledon margins, suppressed *PP2C49* expression, and enhanced *HKT1;1* accumulation in the radicle, collectively supporting improved Na^+^/K^+^ balance. Transcriptome profiling further revealed additional Ca^2+^‐responsive *PP2C* genes under CAMTA6‐dependent regulation. Together, these findings establish a Ca^2+^‐regulated transcriptional network coordinating ion homeostasis during germination and suggest strategies to support germinating embryo performance in saline environments.

## Significance Statement

Salinity impairs germination largely by disrupting Na^+^/K^+^ homeostasis, yet the signaling pathways that protect embryos at this stage remain poorly defined. We identify a calcium‐responsive regulatory mechanism that spatially coordinates transcriptional control of genes involved in ion transport during early development, providing a mechanistic basis for improving seedling establishment in saline soils.

## INTRODUCTION

Salt stress is a major abiotic factor limiting plant growth and productivity, particularly during seed germination and early seedling establishment (Munns & Tester, 2008). The increasing salinization of agricultural soils worldwide (Hassani et al., 2021; Wang et al., 2003) highlights the need for a deeper understanding of the mechanisms governing plant responses to salinity, particularly during the early stages of development. During seed germination, salt stress promotes osmotic stress, disrupting water uptake and seed imbibition, as well as oxidative stress, leading to the accumulation of reactive oxygen species that damage cellular structures. In addition, high salinity results in ionic imbalances, primarily through excessive accumulation of sodium (Na^+^), which impairs metabolic processes that are necessary for germination and seedling growth (Munns & Tester, 2008; Zhu, 2002).

In contrast to Na^+^, potassium (K^+^) is an essential macronutrient that plays a vital role in various physiological processes during seed germination, including enzyme activation, osmoregulation, and maintenance of cellular turgor. Under salt stress, elevated Na^+^ levels can disrupt K^+^ homeostasis, leading to reduced K^+^ uptake and accumulation in germinating embryos. This imbalance adversely affects germination rates and seedling vigor (Sun et al., 2015; Wang et al., 2013; Wang & Wu, 2013).

Plants maintain ion homeostasis under salinity primarily by limiting shoot Na^+^ accumulation via HKT‐mediated retrieval from the root xylem (Assaha et al., 2017; Byrt et al., 2014; Houser & Horie, 2010; Mäser et al., 2002; Uozumi et al., 2000). In Arabidopsis, HKT1;1 is essential for Na^+^/K^+^ homeostasis and seedling development (Berthomieu et al., 2003; Davenport et al., 2007; Horie et al., 2006; Mäser et al., 2002). During germination, HKT1;1 is broadly expressed in wild type embryos, whereas CAMTA6‐mediated Ca^2+^ signaling represses this expression and restricts it to the radicle (Chandran et al., 2024; Shkolnik et al., 2019). In addition, PP2C49 negatively regulates HKT1;1 at the post‐translational level and thereby influences Na^+^ allocation during salt stress (Chu et al., 2021). These findings indicate that CAMTA6 and PP2C49 may act in a coordinated regulatory network controlling HKT1;1 function during germination under salinity, but the mechanistic connection between these layers remains unresolved (Chandran et al., 2024; Chu et al., 2021; Shkolnik et al., 2019).

A key component of the plant's adaptive response is the involvement of Ca^2+^‐mediated signaling pathways that decode salt‐induced cytosolic Ca^2+^ transients and modulate gene expression and hormone sensitivity (Dodd et al., 2010). Ca^2+^ sensors such as calcineurin B‐like proteins (CBLs), CBL‐interacting protein kinases, calmodulin‐like proteins, and Ca^2+^‐dependent protein kinases integrate salt stress signals with abscisic acid (ABA) and gibberellin pathways to inhibit germination (Pandey et al., 2004; Yip Delormel & Boudsocq, 2019). This tightly regulated signaling network ensures that germination proceeds only under favorable conditions.

CAMTAs are key regulators of plant responses to environmental stress, integrating Ca^2+^ signaling with the regulation of gene expression. In Arabidopsis, the CAMTA family consists of six members (CAMTA1–CAMTA6), which participate in diverse physiological processes, including adaptation to both abiotic and biotic stresses (Finkler, Ashery‐Padan, & Fromm, 2007; Galon et al., 2010; Rahman et al., 2016). CAMTAs are known to mediate plant responses to cold, drought, and salinity by modulating stress‐responsive gene expression, particularly through interactions with Ca^2+^/calmodulin signaling cascades (Kim et al., 2017; Yang et al., 2012). Among them, CAMTA6 has emerged as an essential regulator in salt stress adaptation, particularly during seed germination, where it functions as a negative regulator of salt tolerance (Shkolnik et al., 2019). Mutants lacking functional CAMTA6 exhibit enhanced germination rates under high‐salinity conditions, suggesting that the absence of CAMTA6 enhances seed germination by improving Na^+^/K^+^ homeostasis (Chandran et al., 2024). Understanding CAMTA6‐mediated transcriptional regulatory networks can offer valuable insights into the regulation of germination under challenging environmental conditions.

Protein phosphatase 2C (PP2C) proteins play essential roles in abiotic stress signaling, including responses to salt stress during seed germination (Chandran et al., 2024; Park et al., 2009; Schweighofer et al., 2004). In the Arabidopsis genome, more than 80 *PP2C* genes have been identified. Based on sequence similarity and domain structure organization, these *PP2C*s are classified into 10 distinct groups, designated A through J (Schweighofer et al., 2004). Group A PP2Cs, including ABI1, ABI2, HAB1, and HAB2, function as negative regulators of ABA signaling, a key pathway involved in stress responses (Fujii et al., 2009). These phosphatases dephosphorylate and inactivate SNF1‐related protein kinase 2 enzymes, thereby suppressing ABA‐mediated stress responses and adaptation. During seed germination under salt stress, ABA levels increase to germination‐arresting concentrations, and PP2Cs play a crucial role in modulating the balance between germination and dormancy by controlling ABA sensitivity (Antoni et al., 2012; Park et al., 2009). PP2C49 is a group G‐type PP2C that has been implicated in the dephosphorylation of the sodium transporter HKT1;1, thereby controlling ion fluxes and contributing to the maintenance of cellular ion homeostasis under salt stress (Chandran et al., 2024; Chu et al., 2021; Shkolnik et al., 2019). Understanding the role of PP2Cs in salt stress responses, whether through Ca^2+^‐dependent or independent pathways, may provide valuable insights into plant‐resilience mechanisms and identify potential targets for enhancing salt tolerance.

Given the growing challenges posed by global climate change and the progressive salinization of agricultural soils, understanding the molecular, cellular, and physiological mechanisms that regulate plant responses to salinity during germination is essential. Such insights are essential for guiding breeding strategies and biotechnological interventions aimed at boosting salt tolerance in crop species.

## RESULTS

### Ca^2+^‐mediated modulation of CAMTA6, HKT1;1, and PP2C49 expression during salt stress in germinating Arabidopsis

**Figure 1:**
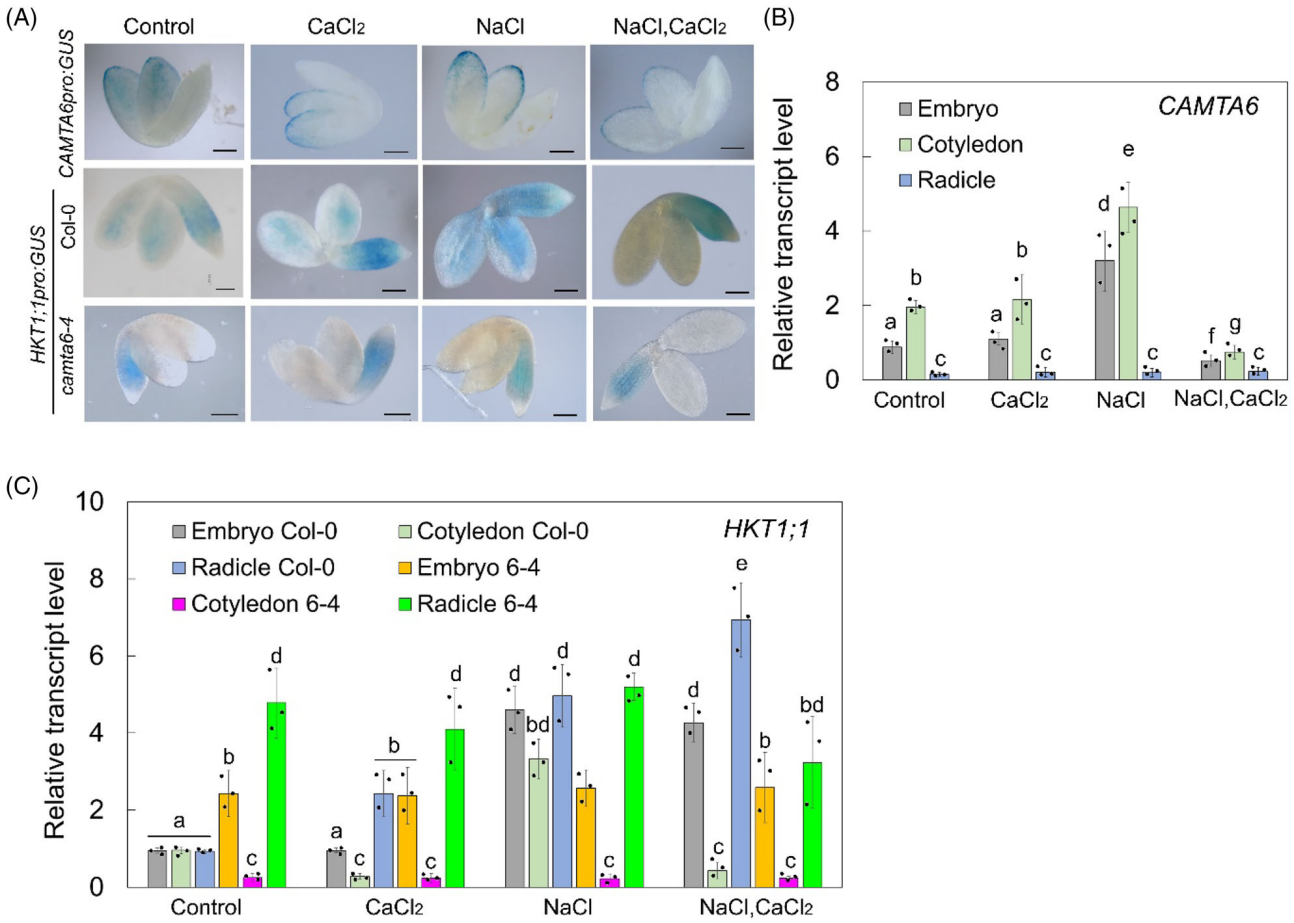
NaCl and CaCl_2_ modulate *CAMTA6* and *HKT1;1* expression in wild type and *camta6‐4* Arabidopsis germinating embryos. (A) GUS staining of ejected embryos harboring *CAMTA6*_*pro*_ *:GUS* or *HKT1;1*_*pro*_ *:GUS* and treated with single or combined chemicals, as indicated (NaCl 150 mM; CaCl_2_ 10 mM) for 16 h before ejection, staining, and imaging (bars = 0.5 mm). (B, C) RT‐qPCR quantification of relative transcript levels of CAMTA6 (B) and HKT1;1 (C) in germinating embryos. Data are presented as means ± SD from three independent biological experiments (30 seeds each). Different lowercase letters indicate significantly different values by Tukey's HSD post hoc test (*P* < 0.01). Individual data points for each replicate are shown as dots overlaid on the box plots.

In germinating Arabidopsis embryos, CAMTA6 is predominantly expressed in the cotyledons, whereas HKT1;1 is expressed in both the cotyledons and the radicle (Chandran et al., 2024; Shkolnik et al., 2019). We previously reported that CaCl_2_ treatment induces radicle‐enriched HKT1;1 expression, correlating with enhanced salt tolerance during germination (Chandran et al., 2024). To further explore how Ca^2+^ and salt stress affect the spatial expression of these genes, we examined *CAMTA6* and *HKT1;1* promoter activity in transgenic lines expressing GUS under the control of each promoter (*CAMTA6pro:GUS* and *HKT1;1pro:GUS*) (Shkolnik et al., 2019). Under all tested conditions, *HKT1;1* promoter activity was absent from the cotyledon margins. In contrast, *CAMTA6* expression became focused or markedly enhanced in these margins following CaCl_2_ or NaCl treatment (Figure 1A). Notably, under combined NaCl and CaCl_2_ treatment, *CAMTA6* promoter activity was observed mainly in the cotyledon margins, whereas *HKT1;1* promoter activity was detected predominantly in the radicle (Figure 1A), consistent with differential promoter activity across embryo tissues under stress.

Similarly, in the *camta6‐4* mutant background, *HKT1;1* expression was confined to the radicle (Figure 1A), indicating that both conditions are associated with a similar reporter pattern of this gene. To validate the spatial expression patterns of *CAMTA6* and *HKT1;1*, we performed reverse transcription quantitative PCR (RT‐qPCR) on RNA extracted from whole embryos, cotyledons, and radicles (see Materials and Methods). Consistent with the promoter activity data, *CAMTA6* transcripts were predominantly detected in cotyledons, with substantially lower expression in radicles under all treatments (Figure 1B). Upon NaCl exposure, *CAMTA6* expression in the cotyledons increased substantially (4.63 ± 0.06, relative to whole embryos set to 1.0), whereas expression in the radicle remained low, albeit evident (0.2 ± 0.1) (Figure 1B). Under the combined CaCl_2_–NaCl treatment, cotyledon *CAMTA6* transcript levels (1.52 ± 0.18) were reduced relative to CaCl_2_ alone (2.16 ± 0.15) and untreated controls (1.96 ± 0.18) (Figure 1B). Under the same combined treatment, *HKT1;1* transcript was strongly enriched in the radicle (6.93 ± 0.96) and low in the cotyledons (0.43 ± 0.2) (Figure 1C). In the *camta6‐4* mutant, *HKT1;1* expression remained confined to the radicle under all tested conditions, consistent with the promoter activity results (Figure 1A,C). Overall, the reporter and RT‐qPCR data are consistent with differential tissue‐associated expression patterns, with *CAMTA6* and *HKT1;1* coordinated by Ca^2+^ across different tissues, which becomes more pronounced under salt stress.

**Figure 2:**
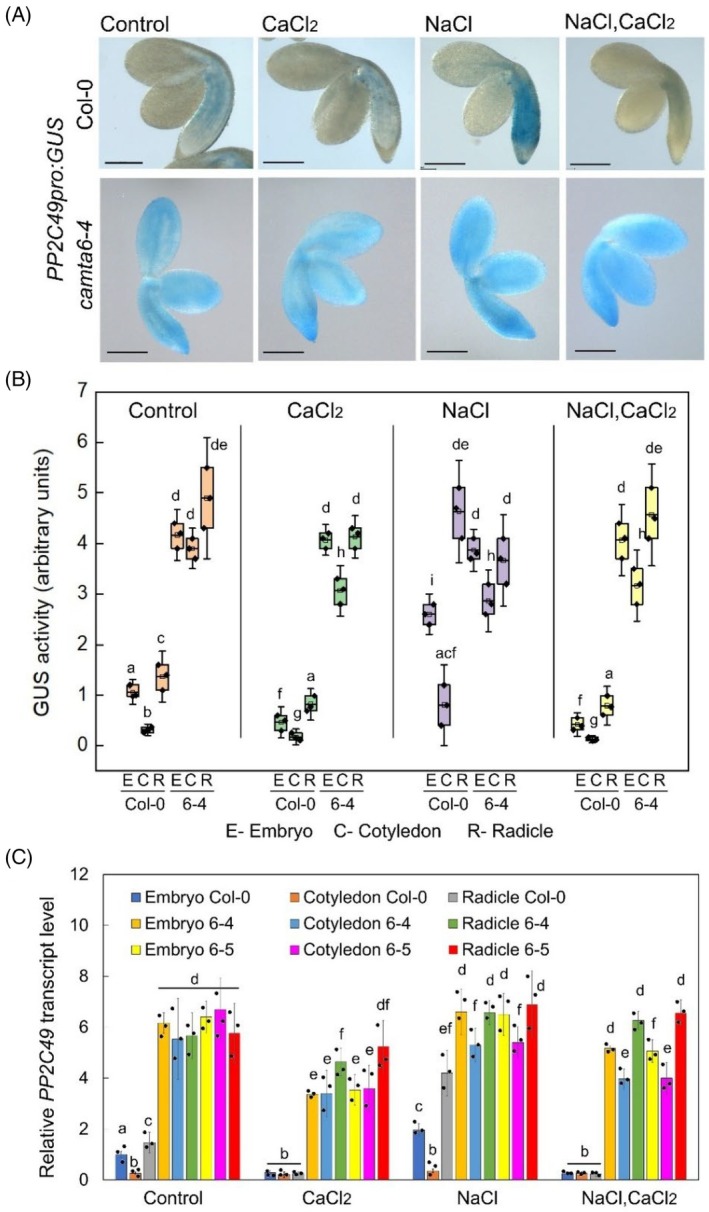
NaCl and CaCl_2_ influence *PP2C49* expression in wild type, while *camta6* mutants exhibit constitutively high expression levels. (A) GUS staining of ejected embryos harboring *PP2C49*_*pro*_ *:GUS* and treated with single or combined chemicals as indicated (NaCl 150 mM; CaCl_2_ 10 mM) for 16 h before ejection, staining, and imaging (bars = 0.5 mm). (B) Spectrophotometric GUS assay. Relative GUS activity of whole root tissue extracts was quantified using PNPG as a substrate. Data are presented as relative units normalized to the control embryo value, which was set to 1. Bold lines in each box indicate the mean, and whiskers represent ± SD. Top and bottom sides of the boxes correspond to the third and first quartiles, respectively. Individual data points for each replicate are shown as dots overlaid on the box plots. (C) RT‐qPCR quantification of relative *PP2C49* transcript levels in germinating embryos. Data are presented as means ± SD. For both (B) and (C), three independent biological experiments (30 seeds each). Different lowercase letters indicate significantly different values by Tukey's HSD post hoc test (*P* < 0.005 for B; *P* < 0.01 for C). Individual data points for each replicate are shown as dots overlaid on the bars.

*HKT1;1* is regulated transcriptionally by CAMTA6 (Shkolnik et al., 2019) and post‐translationally inactivated by PP2C49 through direct dephosphorylation (Chu et al., 2021). We recently showed that *PP2C49* is expressed in the radicle and that CaCl_2_ treatment suppresses its NaCl‐induced expression (Chandran et al., 2024). In the current study, we investigated the transcriptional regulation of *PP2C49* by CAMTA6 by examining the *PP2C49pro:GUS* reporter construct in both wild type and the *camta6‐4* mutant background. While GUS activity in wild type was primarily restricted to the radicle, this repression was abolished in the *camta6‐4* background, where promoter activity was detected across all embryo tissues under all tested conditions (Figure 2A).

To further validate these findings *in planta* and quantify these tissue‐specific patterns, we measured GUS activity in microdissected cotyledons and radicles (Figure 2B). In wild type embryos expressing the *PP2C49pro:GUS* construct, a low basal level of expression was detected in the cotyledons, which increased slightly under NaCl treatment. In contrast, the *camta6‐4* mutant exhibited a significant and uncontrolled increase in GUS activity in both the cotyledons and the radicle across all conditions. These results are consistent with CAMTA6‐dependent repression in both the quantitative GUS assays (Figure 2B) and the RT‐qPCR analysis of endogenous *PP2C49* transcript levels (Figure 2C). These results are consistent with a role for CAMTA6 in restricting the *PP2C49* promoter activity and prevent its ectopic expression outside the radicle, particularly under salt stress.

At later developmental stages (4‐ to 11‐day‐old seedlings), *camta6* mutants exhibit hypersensitivity to salt stress (Shkolnik et al., 2019). To further explore the roles of CAMTA6, PP2C49, and HKT1;1 in the Ca^2+^‐mediated response to NaCl, we examined their expression patterns in 5‐day‐old whole seedlings and their primary root tips following treatment with CaCl_2_, NaCl, or both. All three genes were expressed in the root vasculature, with enhanced promoter activity observed at the hypocotyl–root junction (Figure S1). *CAMTA6* expression was induced by each treatment—CaCl_2_, NaCl, and their combination, whereas *HKT1;1* expression was only induced by the combined treatment, and this induction was absent in the *camta6‐4* background (Figure S1). *PP2C49* expression was induced by NaCl, but this induction was suppressed by CaCl_2_, which counteracted the NaCl effect. In the *camta6‐4* mutant, *PP2C49* promoter activity was highly responsive to NaCl, and the antagonistic effect of CaCl_2_ was retained (Figure S1). Notably, *PP2C49* was also expressed in the root tip columella cells, where its expression was induced by CaCl_2_, NaCl, and their combination, as previously observed during germination (Figure 2A; Figure S1B). These findings are consistent with differential promoter activity across tissues within the resolution of the assays. CAMTA transcription factors localize to the nucleus and bind specific *cis*‐elements in target gene promoters, including the *ABRE/CAMTA*‐binding motif (CACGTG[C/T/G]) and its coupling element *ABRE*‐CE ([C/A]ACGCG[T/C/G]) (Kaplan et al., 2006; Whalley & Knight, 2013). In *HKT1;1*, mutation of an *ABRE* element (ACGTGT) located 75 bp upstream of the translation start codon abolishes its promoter activity (Shkolnik et al., 2019). An additional *ABRE* motif was identified at position −283 bp (Figure S2). However, direct binding of CAMTA6 to the *HKT1;1* promoter has not yet been demonstrated.

We therefore screened the *PP2C49* promoter for similar *cis*‐elements and identified two canonical *ABRE/CAMTA*‐binding motifs (CACGTGTC) at −388 and −287 bp, as well as a full *ABRE*‐CE (CACGCGGC) at −203 bp relative to the translation start codon (Figure S2). To assess direct binding, we performed a promoter activation assay by transiently co‐expressing the GUS reporter gene driven by the HKT1;1 or PP2C49 promoter, along with the CAMTA6 DNA‐binding domain (CAMTA6_CG‐1_) (da Costa Silva, 1994) in *Nicotiana benthamiana* leaves (Figure S3). Constitutive expression of CAMTA6_CG‐1_ led to enhanced GUS activity driven by both promoters (Figure S3A), supporting the idea that the CAMTA6 DNA‐binding domain can associate with promoter *cis*‐elements capable of driving reporter activation. CAMTA6_CG‐1_ expression was verified by RT‐qPCR (Figure S3B). As a control, we tested the promoter of PP2CG1 (AT2G33700), an ABA‐regulated phosphatase that enhances salt tolerance in young Arabidopsis seedlings (Liu et al., 2012), which is phylogenetically closely related to PP2C49 (as detailed below), and observed no activation by CAMTA6_CG‐1_ (Figure S3A,E). Although the *PP2CG1* promoter contains two *ABRE/CAMTA* motifs at −889 and −1089 bp, their distal location may render them ineffective for CAMTA6‐mediated regulation (Figure S2). Importantly, CAMTA6_CG‐1_ contains only the DNA‐binding and recognition domain of CAMTA6, but lacks additional regions required for transcriptional repression. Therefore, this assay reports promoter activation consistent with CAMTA6 binding to promoter *cis*‐elements, rather than the full regulatory behavior of the native CAMTA6 protein, and only increased GUS activity was expected and observed.

### Sanguinarine treatment enhances seed germination under salt stress conditions

**Figure 3:**
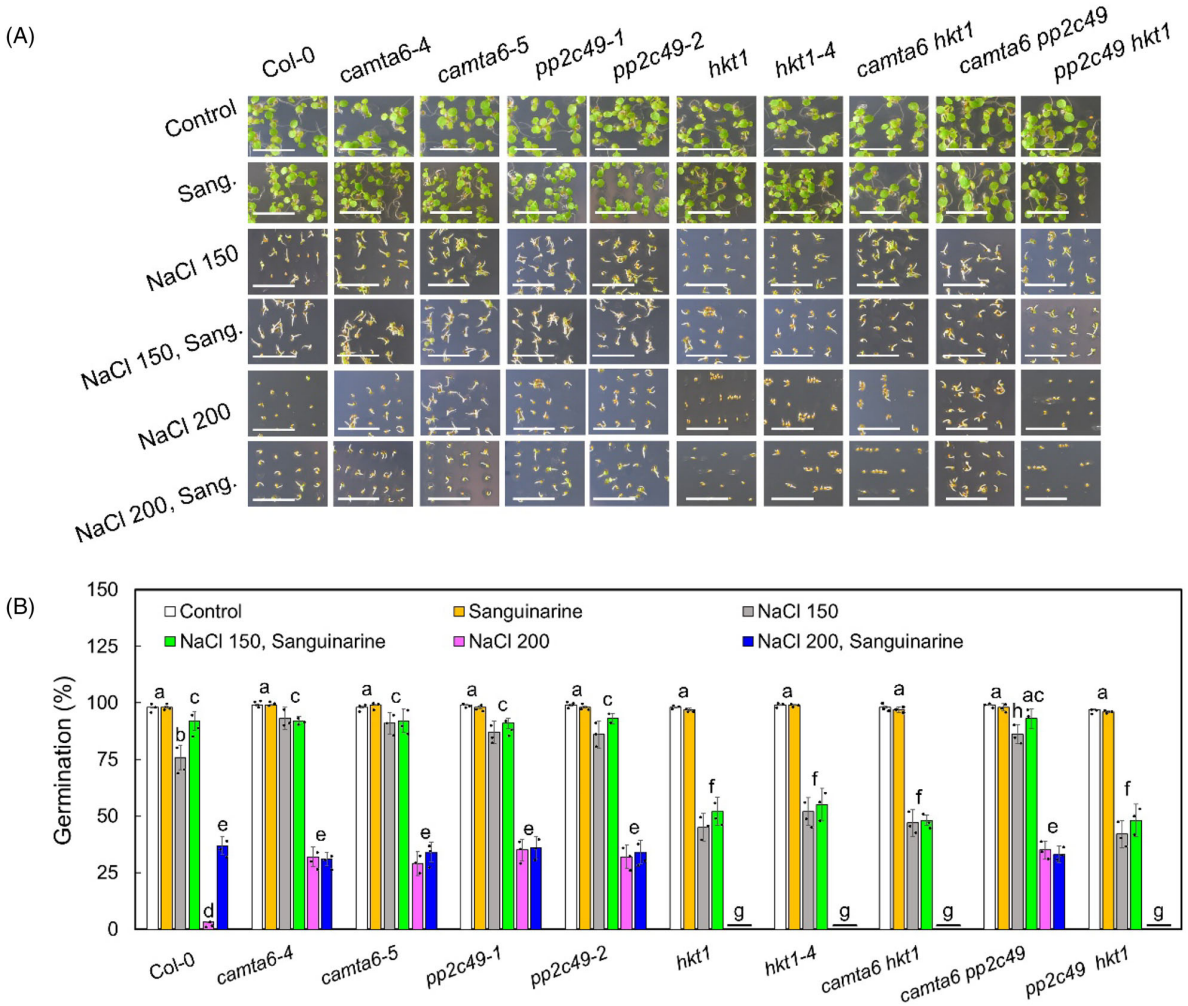
Sanguinarine enhances Arabidopsis seed germination under salt stress. (A) Representative photographs of germinating seedlings sown on medium supplemented with the indicated single or combined chemicals (NaCl, 150 mM or 200 mM; sanguinarine, 1 μM) 5 days after plating (bars = 5 mm). (B) Germination rates of the indicated genotypes. Data are presented as means ± SD from three independent biological experiments (~100 seeds each). Individual data points overlaid on the bars represent the mean value of technical triplicates for each biological replicate. Different lowercase letters indicate significantly different values by Tukey's HSD post hoc test (*P* < 0.001).

Since PP2C49 is known to be involved in salt stress responses during germination (Chandran et al., 2024), we examined whether treatment with sanguinarine, a benzophenanthridine alkaloid and mammalian PP2C inhibitor (Aburai et al., 2010; Vogt et al., 2005), modulates the germination of Arabidopsis seeds under salinity. Supplementation with 1 μM sanguinarine significantly increased wild type germination from 76 to 92% under 150 mM NaCl, and from 3 to 37% under 200 mM NaCl. In contrast, this rescue was compromised in camta6, pp2c49, and hkt1 single and double mutants (Figure 3). Specifically, the loss of HKT1;1 in *camta6 hkt1* and *pp2c49 hkt1* lines prevented any sanguinarine‐induced tolerance, suggesting that the protective effect depends on the CAMTA6‐PP2C49‐HKT1;1 module.

To investigate whether sanguinarine targets canonical group A PP2Cs, key negative regulators of ABA signaling (Antoni et al., 2012; Umezawa et al., 2009), we assessed its impact on germination under exogenous ABA. While ABA inhibited wild type germination in a dose‐dependent manner, 1 μM sanguinarine failed to alleviate this arrest (Figure S4). Furthermore, unlike *camta6* (Shkolnik et al., 2019), both *pp2c49* and *hkt1* mutants exhibited wild type‐like ABA sensitivity when tested across a range of concentrations from 0 to 1000 nM (Figure S5). These results suggest that PP2C49 and HKT1 do not substantially contribute to canonical ABA signaling under the conditions tested, reinforcing that their roles in salt tolerance are ABA independent.

To further explore the role of group G‐type PP2Cs under salt stress, we examined PP2CG1, a close phylogenetic relative of PP2C49 (discussed below as a functional reference to test for conserved activity). Seeds of *pp2cg1* mutants did not exhibit salt tolerance comparable to that of *pp2c49* mutants. Furthermore, sanguinarine treatment enhanced the germination of *pp2cg1* mutants under salt stress, similar to its effect on wild type seeds (Figure 3; Figure S6). These findings tentatively suggest some specificity of sanguinarine toward PP2C49, although effects on other phosphatases cannot be excluded.

To examine how PP2C49 and sanguinarine influence CAMTA6 and HKT1;1 during germination, we utilized promoter‐GUS lines and RT‐qPCR in wild type and *pp2c49‐1* backgrounds. In wild type embryos, sanguinarine reduced CAMTA6 transcript levels and *CAMTA6pro:GUS* activity. Interestingly, CAMTA6 expression was constitutively lower in *pp2c49‐1* and primarily restricted to cotyledon margins (Figure S7A,B). Conversely, sanguinarine increased *HKT1;1* expression across wild type tissues. In the *pp2c49‐1* background, *HKT1;1* transcript and promoter activity were higher than wild type under control conditions, remaining elevated regardless of sanguinarine treatment (Figure S7A,C). These results suggest that both PP2C49 deficiency and sanguinarine application shift the CAMTA6–HKT1;1 balance toward increased HKT1;1 expression.

### Modulation of CAMTA6, PP2C49, and HKT1;1 expression by sanguinarine during salt‐stressed germination

**Figure 4:**
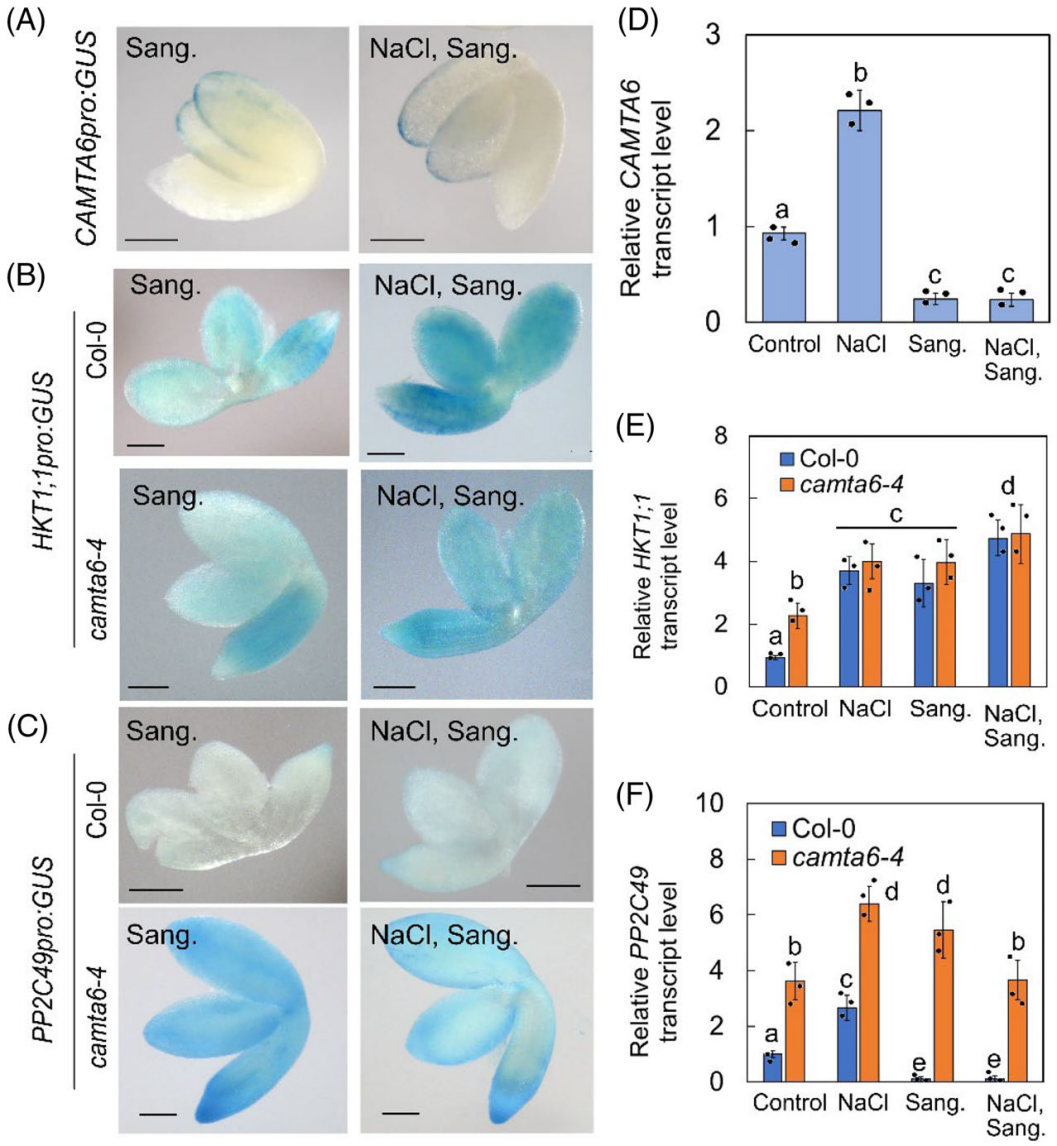
Sanguinarine and NaCl influence *CAMTA6*, *PP2C49*, and *HKT1;1* expression in germinating Arabidopsis embryos, as revealed by GUS staining and RT‐qPCR. Sanguinarine (Sang.) modulates the expression of *CAMTA6*, *PP2C49*, and *HKT1;1* in germinating Arabidopsis. (A–C) GUS staining of ejected wild type (Col‐0) or *camta6‐4* embryos harboring the indicated constructs and treated with 150 mM NaCl and/or 1 μM sanguinarine, as indicated (bars = 0.5 mm). (D–F) RT‐qPCR quantification of relative transcript levels of *CAMTA6* (D), *HKT1;1* (E), and *PP2C49* (F) in germinating embryos. Data are means ± SD (three biological experiments, ~30 seeds each) and different lowercase letters indicate significantly different values by Tukey's HSD post hoc test (*P* < 0.01). Individual data points overlaid on the bars represent the mean value of technical triplicates for each biological replicate.

To further examine the response of CAMTA6, PP2C49, and HKT1;1 to sanguinarine during salt‐stressed germination, we examined the expression patterns of these genes in germinating embryos treated with NaCl and/or sanguinarine. Notably, sanguinarine reduced *CAMTA6* promoter activity, which under control conditions was broadly detected across the cotyledon, but after treatment was observed mainly in the margins (Figures 1A and 4A). The NaCl‐induced increase in CAMTA6 expression was suppressed by sanguinarine treatment (Figures 1A and 4A). Moreover, sanguinarine treatment increased HKT1;1 transcript levels and strongly reduced PP2C49 expression in wild type embryos. Notably, these effects were not observed in the *camta6‐4* mutant background (Figure 4B,C,E,F).

These results were corroborated by RT‐qPCR analysis of whole germinating embryos, revealing mean ± SD relative *CAMTA6* transcript levels of 0.92 ± 0.07 (control), 2.21 ± 0.21 (NaCl), 0.24 ± 0.06 (sanguinarine), and 0.23 ± 0.07 (NaCl–sanguinarine) (Figure 4D). These data are consistent with repression of *CAMTA6* expression by sanguinarine during germination, including under salt stress.

Interestingly, although *camta6* mutants germinate more efficiently under salt stress, they are hypersensitive at later seedling stages (Shkolnik et al., 2019). Unlike seed germination, sanguinarine treatment enhanced *CAMTA6* promoter activity in the root vasculature of young seedlings, resembling the response to CaCl_2_, NaCl, and their combined application (Figure S8). In wild type seedlings, NaCl treatment induced the expression of both *HKT1;1* and *PP2C49*, whereas sanguinarine suppressed both transcripts. Notably, the NaCl‐induced upregulation of *PP2C49*, but not *HKT1;1*, was attenuated by sanguinarine. In the *camta6‐4* background, the responsiveness of the HKT1;1 and PP2C49 promoters to these treatments was largely similar to that observed in wild type seedlings. However, the strong NaCl‐induced activation of the PP2C49 promoter in young root zones, and its repression by sanguinarine, persisted in the *camta6‐4* mutant, suggesting the involvement of a CAMTA6‐independent regulatory component (Figure S8).

To determine if sanguinarine's protective effect extends beyond germination, 3‐day‐old seedlings were transferred to media containing NaCl, sanguinarine, or both. While sanguinarine promotes germination rates under salinity (Figure 3), it failed to mitigate salt‐induced reductions in primary root elongation or chlorophyll retention in established seedlings (Figure S9). Consistent with previous reports, *camta6* and *hkt1* mutants exhibited their characteristic salt hypersensitivity (Mäser et al., 2002; Shkolnik et al., 2019). These results indicate that sanguinarine's benefits are stage‐specific and do not sustain physiological vigor in young seedlings under saline conditions (Figure S9B,C).

To examine whether sanguinarine inhibits PP2C enzymatic activity in plants, we measured phosphatase activity in crude root protein extracts of 4‐day‐old Arabidopsis seedlings using para‐nitrophenyl phosphate (pNPP) as substrate. Sanguinarine significantly reduced phosphatase activity in Col‐0 extracts, from 1.00 ± 0.10 to 0.16 ± 0.04 (arbitrary fluorescence units, a.u.). In contrast, extracts from *pp2c49‐1* roots displayed substantially lower basal phosphatase activity compared with wild type (0.33 ± 0.09 a.u.); this residual activity was further reduced by sanguinarine treatment, though to a much lesser extent than in wild type (0.09 ± 0.03 a.u.) (Figure S10). Together, these data indicate that sanguinarine inhibits phosphatase activity in Arabidopsis roots, with reduced sensitivity observed in the *pp2c49–1* mutant.

### Sanguinarine enhances K^+^ accumulation in germinating embryos under salt stress conditions

**Figure 5:**
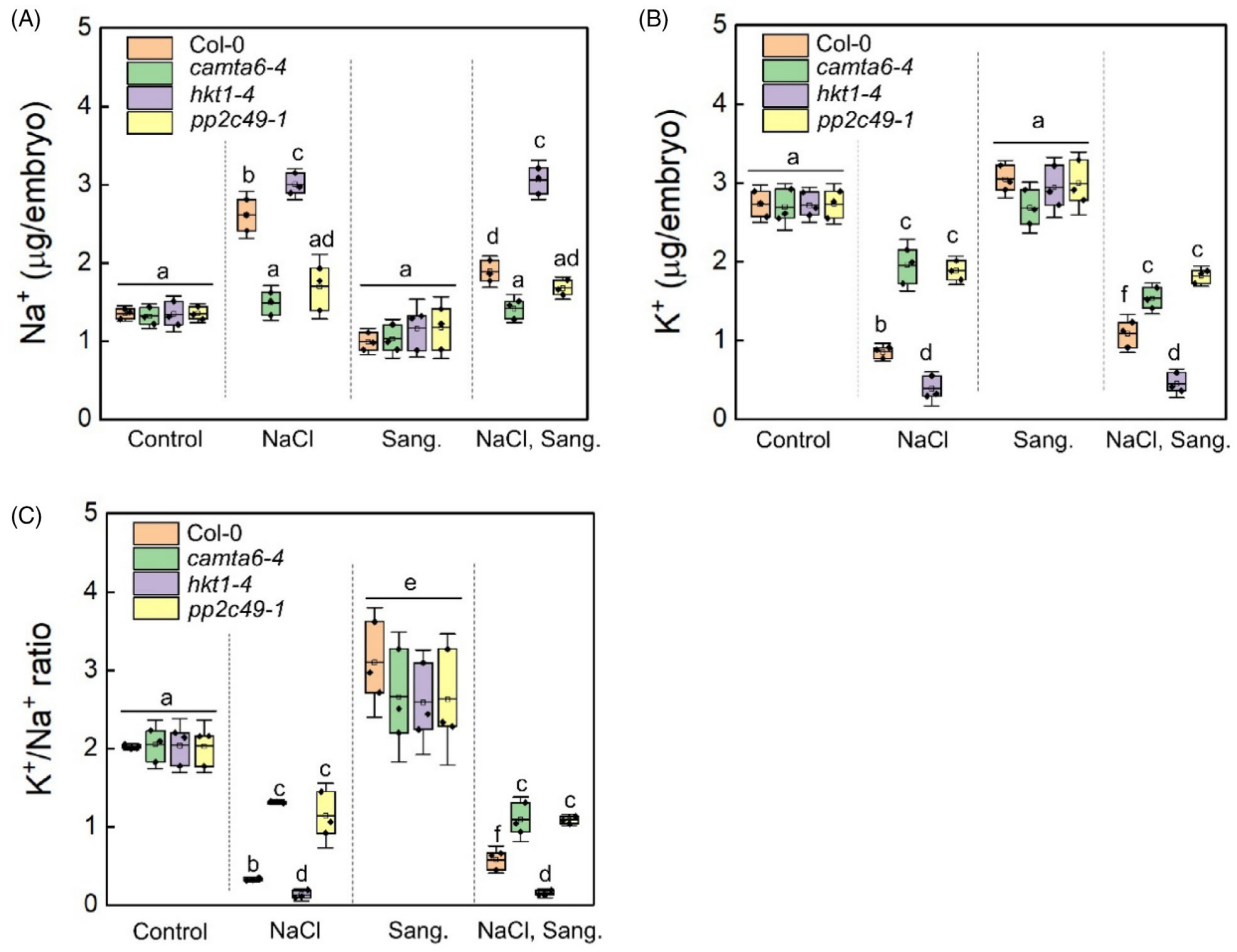
Accumulation of Na^+^ and K^+^ in germinating Arabidopsis embryos under NaCl and sanguinarine treatments. Accumulation of Na^+^ (A) and K^+^ (B), and K^+^/Na^+^ ratios (C) in wild type (Col‐0), *camta6‐4*, *pp2c49‐1*, and *hkt1‐4* germinating embryos in the presence of NaCl and/or sanguinarine (Sang.). Ion content was determined by ICP‐OES. Seeds were treated with 0 (control), 1 μM Sang., 150 mM NaCl, or both, as indicated, for 16 h before embryo ejection. Data are presented as means ± SD from three independent biological experiments (~40 seeds each). Different lowercase letters indicate significantly different values by Tukey's HSD post hoc test (*P* < 0.01). Individual data points for each replicate are shown as dots overlaid on the box plots.

Under salt stress, K^+^ accumulation is inhibited and is associated with arrest of the seed germination process (Chandran et al., 2024; Shkolnik et al., 2019). Supplementing the growth medium with CaCl_2_ has been shown to enhance K^+^ accumulation and improve germination rates under salt stress (Chandran et al., 2024). In this study, compared to wild type embryos, germinating *hkt1* mutants accumulate higher Na^+^ and lower K^+^ levels under salt stress, whereas *pp2c49* and *camta6* mutants exhibit the opposite ion profile under the same conditions (Figure 5). Based on these findings, we investigated whether sanguinarine influences Na^+^ and K^+^ contents in germinating wild type, *camta6*, *hkt1*, and *pp2c49* mutants in the presence of NaCl.

To quantify Na^+^ and K^+^ accumulation in germinating wild type, *camta6‐4*, *hkt1‐4*, and *pp2c49‐1* embryos, we analyzed embryo extracts using inductively coupled plasma optical emission spectrometry (ICP‐OES) as previously described (Shkolnik et al., 2019). Seeds were treated for 16 h with 1 μM sanguinarine, 150 mM NaCl, both, or no treatment (control). In response to NaCl, wild type, *camta6‐4*, *hkt1‐4*, and *pp2c49‐1* embryos accumulated 2.61 ± 0.2, 1.49 ± 0.14, 3.10 ± 0.13, and 1.7 ± 0.26 μg Na^+^ per embryo, respectively (Figure 5), consistent with the previously reported reduced Na^+^ accumulation in *camta6* and *pp2c49* embryos under salt stress (Chandran et al., 2024; Shkolnik et al., 2019).

Notably, in response to the combined NaCl–sanguinarine treatment, wild type embryos accumulated 1.89 ± 0.13 μg Na^+^ per embryo, a substantial reduction compared to NaCl treatment alone. In contrast, *hkt1‐4* embryos accumulated 3.06 ± 0.17 μg Na^+^ per embryo, similar to their response to NaCl alone (Figure 5). Similarly, *camta6‐4* and *pp2c49‐1* embryos showed no significant difference in Na^+^ accumulation between NaCl and the combined treatment, with 1.41 ± 0.12 μg and 1.67 ± 0.10 μg Na^+^ per embryo, respectively (Figure 5). Under control and sanguinarine‐only conditions, all genotypes accumulated comparable Na^+^ levels, approximately 1.32–1.35 μg and 0.99–1.17 μg per embryo, respectively (Figure 5).

Analysis of K^+^ concentrations in response to NaCl and NaCl–sanguinarine treatments revealed accumulation levels of 0.85 ± 0.07 μg and 1.10 ± 0.16 μg per embryo in the wild type, 1.95 ± 0.21 μg and 1.53 ± 0.13 μg in *camta6‐4*, 0.38 ± 0.14 μg and 0.45 ± 0.12 μg in *hkt1‐4*, and 1.90 ± 0.12 μg and 1.81 ± 0.08 μg in *pp2c49‐1*, respectively (Figure 5). Under control and sanguinarine‐only treatments, all genotypes accumulated similar amounts of K^+^, ranging from ~2.70–2.73 μg and ~2.68–3.10 μg per embryo, respectively (Figure 5).

### Transcriptome analysis identifies NaCl‐ and CaCl_2_ ‐responsive *PP2C*s modulated by CAMTA6 during Arabidopsis germination

To investigate the extent of PP2C involvement in the germinating embryo response to salt, Ca^2+^, and CAMTA6 activity, we re‐analyzed two complementary transcriptome datasets. One compared germinating *camta6‐5* mutants to wild type embryos under NaCl stress (Shkolnik et al., 2019; Table S1), and the other profiled wild type responses to NaCl, CaCl_2_, or their combination (Chandran et al., 2024; Table S2). Re‐examining these data suggested that several PP2Cs are salt‐responsive in a CAMTA6‐associated manner.

Cross‐examination of the transcriptome datasets identified 63 *PP2C* genes, including *PP2C49*, as responsive to the tested conditions (Tables S1 and S2). We identified 40 genes that overlapped in the two transcriptome datasets and organized them into a phylogenetic tree consisting of 9 groups (A–H and J) using the Randomized Axelerated Maximum Likelihood (RAxML) and FigTree tools (Figure S11A), as reviewed (Schweighofer et al., 2004). Among them, 12 genes were detected in both datasets and met the selection criteria of fold change greater than 1.25 and *P* < 0.05 in at least one of the examined conditions (Table S3). This relatively permissive threshold was chosen to ensure a comprehensive identification of the regulatory landscape, preventing the exclusion of biologically relevant genes that may exhibit modest but functionally significant expression changes during early stress responses. Among the four group A‐type *PP2C*s identified, *AHG1* and *HAI2* exhibited strong NaCl‐inducible expression in the wild type, with fold changes of 9.35 (*P* = 1.42E^−6^) and 14.97 (*P* = 1.65E^−6^), respectively (Table S3). In the *camta6‐5* mutant, this induction was markedly attenuated, with fold changes reduced to 4.55 (*P* = 2.61E^−5^) and 5.39 (*P* = 5.58E^−5^), reflecting a 35.74% and 64% loss of NaCl‐inducible transcript accumulation, respectively. These findings identify AHG1 and HAI2 as CAMTA6‐associated salt‐responsive candidates. In contrast, the other identified group A *PP2Cs*, *ABI1* and *ABI2*, showed only minor responsiveness to NaCl and were largely unaffected by the *camta6‐5* mutation (Table S3), indicating a CAMTA6‐independent regulatory mechanism.

Of the three group F‐type *PP2C*s identified, the one encoded by AT2G34740 exhibited a prominent NaCl response, with a fold change of 74.75 (*P* = 3.02E^−6^) in the wild type. This induction was substantially reduced by 77.66%, dropping to 16.7 (*P* = 7.02E‐5) in the *camta6‐5* mutant (Table S3). These data strongly suggest a role for this PP2C in the CAMTA6‐modulated germinating embryo's response to salt.

To validate the expression patterns observed in the transcriptome analysis and ensure the reliability of the RNA‐seq data, we performed RT‐qPCR on three representative PP2C genes showing distinct responses to salinity and the *camta6‐5* mutation: *APD8*, *ABI2*, and *PP2C.D6* (Figure S11B). The RT‐qPCR results were highly consistent with the trends identified in the global transcriptomic datasets, confirming the robustness of our analysis. Specifically, APD8 showed a 1.42‐fold induction in response to NaCl in the wild type, whereas in the *camta6‐5* mutant, this induction was significantly more pronounced, reaching a 3.26‐fold increase relative to its respective control (from 0.86 to 2.82). For *ABI2*, expression was strongly induced by salt in both genotypes, with a 3.18‐fold induction in the wild type and a 4.0‐fold induction in *camta6‐5*, indicating a largely CAMTA6‐independent salt response for this gene. Finally, *PP2C.D6* exhibited a moderate but consistent NaCl‐inducible pattern that was similar across both wild type and mutant backgrounds (approximately 1.52‐fold and 1.44‐fold induction, respectively). The close alignment between the RT‐qPCR and RNA‐seq datasets reinforces the validity of our selection criteria, including the 1.25‐fold change cutoff, in identifying biologically relevant CAMTA6‐regulated targets that might otherwise be overlooked at higher thresholds.

To gain a comprehensive overview of the 63 identified *PP2C* genes, we performed a heatmap analysis (Figure S12). This visualization reveals that while a broad range of these genes is downregulated (blue) in response to NaCl and CaCl_2_ in the wild type, this pattern is markedly reversed in the *camta6‐5* mutant, where these clusters become significantly upregulated (red). This indicates that CAMTA6 is required for the proper transcriptional modulation of these *PP2C* members during the response to salt and CaCl_2_, broadening our understanding of how CAMTA6 modulates early responses to salt through selective *PP2C* regulation.

## DISCUSSION

### Ca^2+^ modulates the spatial expression of *HKT1;1* via CAMTA6

Seed germination is a tightly controlled developmental process that is highly sensitive to abiotic stresses, including salinity (Nonogaki et al., 2010). Ca^2+^ signaling plays a central role in mediating adaptive responses during germination under stress conditions (Steinhorst & Kudla, 2013). In this study, we demonstrate that CAMTA6, a Ca^2+^/calmodulin‐dependent transcription factor, modulates seed germination under salt stress conditions, linking Ca^2+^ signaling to ionic homeostasis mechanisms.

Our findings reveal that CAMTA6 modulates the expression of HKT1;1, a Na^+^/K^+^ transporter known to limit sodium accumulation in plant tissues (Sunarpi et al., 2005). Under control conditions, CAMTA6 expression is predominantly confined to the cotyledons of germinating Arabidopsis embryos. Upon salt stress, CAMTA6 expression is markedly enhanced and becomes strongly localized to the cotyledon margins (Figure 1A,B). In contrast, HKT1;1 expression is broadly detected across all embryo tissues under both control conditions and in response to NaCl treatment, except for the cotyledon margins (Figure 1A,C).

This pattern is consistent with a model in which CAMTA6 contributes to the spatial regulation of HKT1;1 expression, particularly at the cotyledon margins, and that its upregulation under salt stress serves to fine‐tune HKT1;1 spatial expression in response to that stress. Upon CaCl_2_ treatment, CAMTA6 expression is reduced and restricted to the cotyledon margins, whereas HKT1;1 expression becomes predominantly localized in the radicle (Figure 1A,C). This pattern is consistent with elevated Ca^2+^ levels reducing *CAMTA6* expression, with *HKT1;1* expression becoming predominantly localized in the radicle, similar to its pattern observed in the *camta6* mutant background (Figure 1A,C).

In the absence of functional CAMTA6, *HKT1;1* expression is primarily observed in the radicle tissues across all tested conditions, a pattern that correlates with enhanced salt tolerance during seed germination (Chandran et al., 2024; Shkolnik et al., 2019). These observations suggest that *HKT1;1* expression may also be regulated by additional factors, potentially including other CAMTA family members or alternative Ca^2+^‐responsive transcriptional regulators capable of recognizing the *ABRE* sequence (Finkler, Kaplan, & Fromm, 2007; Kaplan et al., 2006). In the *camta6‐5* mutant background, *PP2C49* expression was no longer strictly limited to the radicle but showed a wider distribution within the germinating embryo under all tested conditions, indicating a loss of strict spatial regulation. Nonetheless, pronounced expression at the radicle was still evident, as in the wild type. This localized expression (Figure 2A) is consistent with a role for PP2C49 in environmental sensing and in coordinating the transition from seed coat rupture to post‐germination growth. The expanded expression domain in *camta6‐5* implies that CAMTA6 may function to spatially constrain *PP2C49* transcription during early seedling development, potentially linking Ca^2+^ signaling to the spatial control of phosphatase activity.

*camta6* mutants exhibit enhanced tolerance to salinity during germination (Shkolnik et al., 2019), a phenotype that appears to run counter to the observed broad and unregulated expression of *PP2C49* in this background. Given the known inhibitory effect of *PP2C49* on *HKT1;1* function (Chu et al., 2021), such misexpression would be expected to suppress *HKT1;1* activity and increase salt sensitivity. One possible explanation for this discrepancy is that altered spatial regulation impairs the normal function of *PP2C49*, potentially through mislocalization, disruption of interaction networks, or dosage imbalance. Similar cases have been reported for other group A‐type PP2Cs, including *ABI1*, where constitutive or misregulated expression resulted in dominant‐negative effects or altered subcellular dynamics, ultimately compromising phosphatase activity (Merlot et al., 2001; Saez et al., 2004). These observations suggest that precise spatial and temporal control of *PP2C49* expression appears to support its function during germination under saline conditions and that CAMTA6 contributes to this regulatory specificity. The radicle‐enriched expression of *HKT1;1* may enhance salt tolerance during germination by limiting Na^+^ accumulation in the cotyledons, where salt‐sensitive processes such as chloroplast biogenesis are critical for successful germination and the transition to a photosynthetically active seedling (Peharec‐Štefanić et al., 2013). Nevertheless, this hypothetical spatial distribution remains to be directly confirmed by measuring the Na^+^ concentrations specifically in cotyledons of control and salt‐stressed seedlings.

Previous studies have shown that germinating *hkt1* mutants accumulate higher levels of Na^+^ and lower levels of K^+^ under salt stress, compared to wild type seedlings, indicating impaired ion homeostasis (Chandran et al., 2024; Shkolnik et al., 2019). In mature seedlings and adult plants, HKT1;1 is mainly expressed in the vasculature, where it retrieves Na^+^ from the xylem sap to protect the shoot from salt injury (Møller et al., 2009; Sunarpi et al., 2005). By contrast, in germinating seedlings, HKT1;1 expression appears more widespread, including radicle and inner cotyledon tissues, which may facilitate Na^+^ efflux to the medium. Loss of this activity in the *hkt1* mutant is associated with increased Na^+^ accumulation and a pronounced reduction of the K^+^/Na^+^ ratio under salinity stress (0.13 ± 0.05 versus 0.32 ± 0.01 in Col‐0, mean ± SD, Figure 5C; Table 1). Importantly, limiting Na^+^ accumulation is essential to maintain K^+^ homeostasis during early development, a balance critical for seedling establishment under salinity stress (Deinlein et al., 2014).

**Table 1:** Ion content and K^+^/Na^+^ ratios in germinating embryos of Arabidopsis wild type (Col‐0), *camta6‐4*, *pp2c49‐1*, and *hkt1‐4* mutants under salt and/or sanguinarine treatments

Genotype	Treatment	K^+^ (μg/embryo)	Na^+^ (μg/embryo)	K^+^/Na^+^
Col‐0	Control	2.73 ± 0.16	1.35 ± 0.07	2.02 ± 0.02
	NaCl	0.85 ± 0.07	2.61 ± 0.21	0.32 ± 0.01
	Sanguinarine	3.04 ± 0.15	0.99 ± 0.11	3.11 ± 0.46
	NaCl–sanguinarine	1.08 ± 0.16	1.88 ± 0.13	0.58 ± 0.11
*camta6‐4*	Control	2.69 ± 0.19	1.32 ± 0.11	2.05 ± 0.21
	NaCl	1.95 ± 0.21	1.48 ± 0.14	1.31 ± 0.01
	Sanguinarine	2.68 ± 0.21	1.03 ± 0.16	2.65 ± 0.55
	NaCl–sanguinarine	1.53 ± 0.13	1.41 ± 0.12	1.11 ± 0.21
*pp2c49‐1*	Control	2.73 ± 0.17	1.35 ± 0.08	2.03 ± 0.22
	NaCl	1.88 ± 0.12	1.69 ± 0.27	1.14 ± 0.27
	Sanguinarine	2.99 ± 0.26	1.17 ± 0.26	2.62 ± 0.55
	NaCl–sanguinarine	1.81 ± 0.08	1.67 ± 0.09	1.08 ± 0.05
*hkt1‐4*	Control	2.71 ± 0.14	1.34 ± 0.15	2.04 ± 0.22
	NaCl	0.38 ± 0.12	3.10 ± 0.13	0.13 ± 0.05
	Sanguinarine	2.94 ± 0.25	1.16 ± 0.24	2.59 ± 0.44
	NaCl–sanguinarine	0.45 ± 0.12	3.06 ± 0.16	0.15 ± 0.03

Seeds were germinated in the presence of 0 (control), 1 μM sanguinarine, 150 mM NaCl, or both, as indicated. Absolute ion contents (Na^+^ and K^+^) were determined after 16 h of treatment using ICP‐OES, and K^+^/Na^+^ ratios were calculated accordingly. Data represent means ± SD of three biological replicates. The inclusion of absolute ion concentrations highlights the specific effect of sanguinarine on Na^+^ exclusion in the wild type, a response that is abolished in the signaling mutants.

The ability of *camta6‐4* and *pp2c49‐1* to maintain high K^+^/Na^+^ ratios under salt stress underscores the regulatory impact of the CAMTA6‐PP2C49 module. Our model suggests that in these mutants, the removal of negative regulation—at both transcriptional and post‐translational levels—stabilizes HKT1;1 expression in the radicle. This compensatory effect correlates with sustained ion homeostasis, preventing toxic accumulation even under inhibitory treatments that impair the wild type response. Consequently, CAMTA6 and PP2C49 act as critical negative regulators whose absence is associated with enhanced HKT1;1 activity and improved ion homeostasis during germination.

### Ca^2+^ modulation of seed germination under salt stress through PP2Cs

Our findings suggest that PP2C49 functions as a critical link between CAMTA6 and HKT1;1 within the Ca^2+^‐signaling pathway. To further explore this regulatory network, we searched for additional PP2Cs that are responsive to NaCl and Ca^2+^ or exhibit altered expression in the *camta6* mutant background, based on available transcriptome datasets (Chandran et al., 2024; Shkolnik et al., 2019).

Transcriptome analysis revealed that salt stress induces the expression of group A‐type PP2C genes *AHG1* and *HAI2* (Figure S11A; Table S3), both of which are well‐established negative regulators of ABA signaling (Fujii et al., 2009; Park et al., 2009). Interestingly, the salt‐induced expression of *AHG1* and *HAI2* was significantly attenuated in the *camta6‐5* mutant, by 35.7 and 64%, respectively (Table S3) suggesting that CAMTA6 contributes to their transcriptional activation in response to salinity. Given that CAMTAs function as Ca^2+^‐regulated transcription factors implicated in abiotic stress responses (Doherty et al., 2009; Galon et al., 2010), this finding raises the possibility that CAMTA6 integrates Ca^2+^‐ and ABA‐signaling pathways under salt stress. Notably, other group A‐type PP2Cs, such as *ABI1* and *ABI2*, showed only moderate induction by salt, and their expression was largely unaffected in *camta6‐5*, highlighting a selective role for CAMTA6 in modulating a specific subset of ABA‐related stress‐responsive genes.

Among several group F‐type PP2C‐encoding genes identified in our transcriptomic analysis, AT2G34740 exhibited particularly strong induction under salt stress (Table S3), with strong upregulation in both wild type (74.75‐fold) and *camta6‐5* mutant (16.7‐fold) embryos, suggesting that CAMTA6 may contribute to its transcriptional activation under saline conditions. While group A‐type PP2Cs are well‐known negative regulators of ABA signaling (Park et al., 2009; Umezawa et al., 2009), group F‐type members remain less characterized. Nevertheless, functional studies in transgenic tobacco have shown that overexpression of a group F‐type PP2C from wheat can enhance tolerance to salt stress and confer ABA insensitivity (Hu et al., 2015), pointing to roles beyond canonical ABA signaling. The salt‐inducible expression of AT2G34740 during germination, coupled with its reduced induction in the *camta6* mutant, identifies it as a promising candidate for further investigation into the contribution of group F‐type PP2Cs to early stage stress adaptation. Notably, the individual validation of these specific candidates illustrates how targeted RT‐qPCR can support transcriptomic trends, though broader validation of the remaining gene set is required to confirm general regulatory patterns.

Sanguinarine, a plant‐derived benzophenanthridine alkaloid, was found here to enhance salt stress tolerance during seed germination (Figure 3). Based on its reported selective inhibition of PP2Cs in mammalian systems (Aburai et al., 2010), sanguinarine was applied to test its effect on ABA‐mediated inhibition of germination. However, sanguinarine treatment did not alleviate ABA‐mediated inhibition of germination in wild type Arabidopsis seeds (Figure S4). Furthermore, mutants of *PP2C49* and *HKT1;1* displayed germination rates comparable to the wild type across a range of ABA concentrations (Figure S5). These results suggest that PP2C49 and HKT1;1 affect germination through pathways that are largely independent of canonical ABA signaling. The combined lack of ABA‐sensitivity alteration in these mutants and the ineffectiveness of sanguinarine in modulating ABA's inhibitory effect imply that the PP2Cs targeted by sanguinarine are unlikely to be major contributors to ABA‐mediated germination inhibition in Arabidopsis. While further validation of sanguinarine's specificity for plant PP2Cs is warranted, these findings underscore the functional specialization within the PP2C family, supporting the view that only a subset of group A‐type PP2Cs—such as AHG1 and HAI2—function as ABA‐responsive regulators during salt stress, potentially downstream of CAMTA6. It is important to note that PP2C49 belongs to group G‐type of the PP2C family, distinct from group A‐type members such as PP2CA/PPAC, which are well‐established negative regulators of ABA signaling (Park et al., 2009; Umezawa et al., 2009). This classification is consistent with our findings that the salt‐related phenotypes of *pp2c49* and *hkt1* mutants are largely ABA‐independent (Figure S5). The widespread alterations in *PP2C* expression profiles across the *camta6‐5* mutant (Figure S12) further support the role of CAMTA6 as a high‐level orchestrator of the salt and Ca^2+^ response.

Phosphatase activity assays provided biochemical support for the inhibitory effect of sanguinarine on Arabidopsis root PP2Cs. The marked reduction in activity observed in wild type extracts, contrasted with the lower basal activity of *pp2c49‐1* extracts and their reduced response to sanguinarine (Figure S10), is consistent with a functional contribution of PP2C49 to the measured activity. These results suggest that sanguinarine inhibits root‐derived PP2C activity and raises the possibility that PP2C49 is among its primary targets. However, given the large number of PP2Cs expressed in Arabidopsis roots (Xue et al., 2008) and the fact that *pp2cg1* seeds still responded to sanguinarine (Figure S6), these data suggest that PP2C49 is likely among the more relevant targets of sanguinarine, although further work is needed to fully define its specificity.

**Figure 6:**
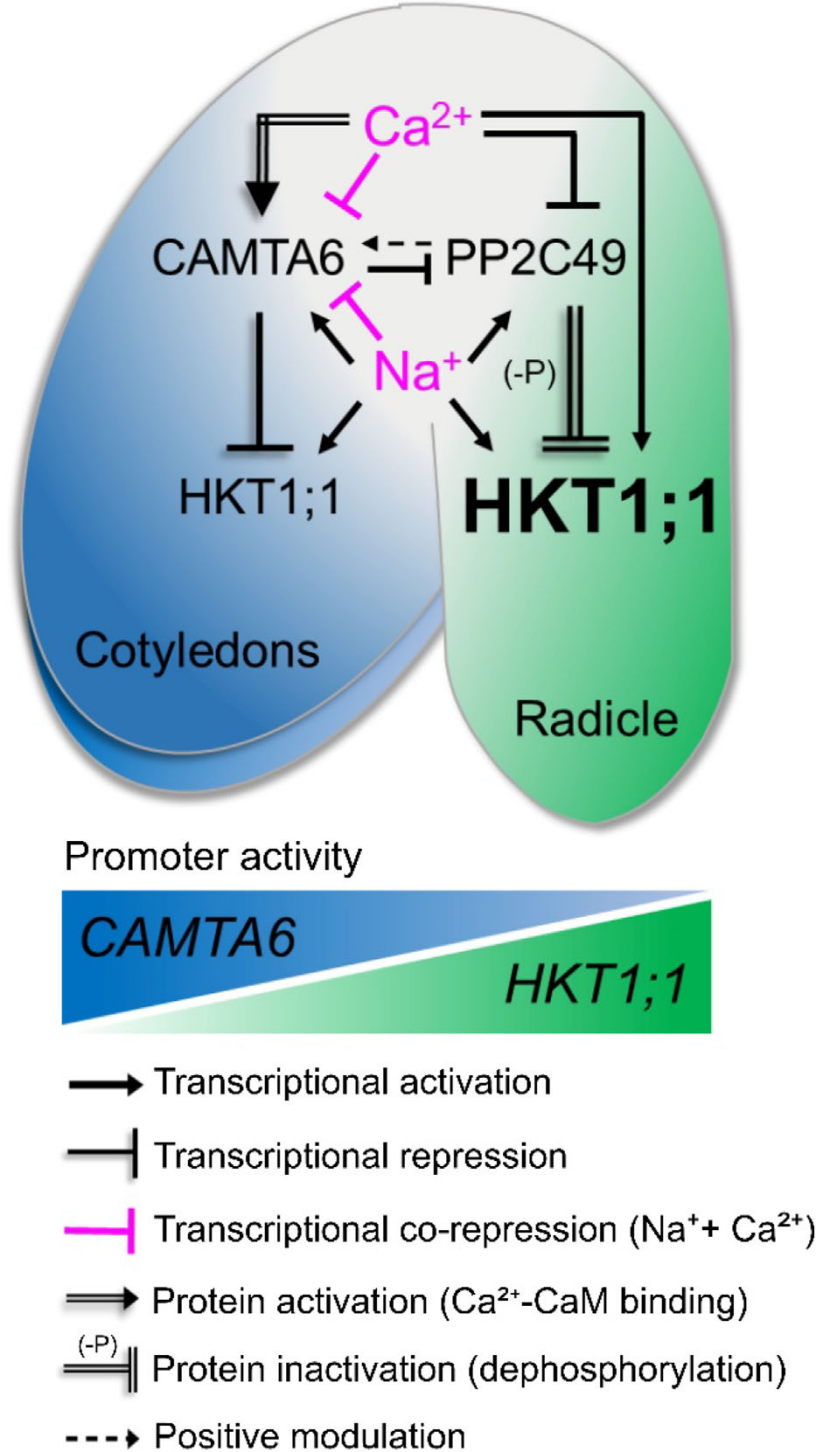
Ca^2+^‐mediated signaling coordinates the expression and activity of CAMTA6, PP2C49, and HKT1;1 to integrate salt stress responses during seed germination. In this model, Ca^2+^ signaling promotes expression of the transcription factor CAMTA6 at the cotyledon margins and activates it via Ca^2+^–CaM binding, induces radicle‐confined expression of the K^+^/Na^+^ symporter HKT1;1, and simultaneously represses its direct negative regulator, the phosphatase PP2C49. PP2C49 appears to inactivate HKT1;1 through direct dephosphorylation (Chu et al., 2021). In contrast, Na^+^ alone enhances CAMTA6 expression at the cotyledon margins, expands HKT1;1 expression throughout the embryo (excluding the margins), and activates PP2C49 specifically in the radicle (Figure 1). Under combined CaCl_2_ and NaCl treatment, CAMTA6 expression is attenuated (Figure 1). The dashed arrow represents positive modulation of PP2C49 by CAMTA6 (see Figure S13).

Our findings suggest a model in which CAMTA6 serves as a spatial and transcriptional rheostat for PP2C49. As proposed in our model (Figure 6; Figure S13), salt stress appears to alter the distribution of CAMTA6 toward cotyledon margins. This shift correlates with strong PP2C49 induction in the radicle, while higher CAMTA6 levels in cotyledons exert dose‐dependent repression. Notably, even the basal CAMTA6 levels in the radicle, detected by RT‐qPCR (Figure 1B), are essential to prevent derepression; thus, in the *camta6‐5* mutant, removing this molecular ‘brake’ leads to the expanded expression observed (Figure 2).

The integration of Ca^2+^ signaling with CAMTA6‐dependent regulation creates an essential potential checkpoint against HKT1;1 over‐inhibition. While direct binding of CAMTA6 to the PP2C49 promoter remains to be demonstrated, our findings support this link as contributing to ion homeostasis. Under salt stress, the enrichment of CAMTA6 at cotyledon margins suggests a localized relief of repression in internal tissues (Figure 1). This spatial regulation may help manage Na^+^ exclusion in the embryo core to protect proplastids and safeguard future chlorophyll synthesis, which is highly sensitive to ionic stress (Turan & Tripathy, 2015), ensuring photosynthetic competence upon germination. Our model (Figure S13) further proposes a feedback loop where PP2C49 promotes CAMTA6 expression, offering a sophisticated mechanism for fine‐tuning salt responses. Finally, given CAMTA6's broad regulatory scope (Shkolnik et al., 2019), it is likely that additional downstream targets also influence PP2C49 and contribute to the global derepression observed in the *camta6* background.

In summary, our results suggest that CAMTA6 mediates responses to salt stress during germination by integrating Ca^2+^ signals into transcriptional regulation, in part through modulation of specific PP2Cs, as CAMTA6 is known to regulate multiple target genes (Shkolnik et al., 2019). This Ca^2+^‐dependent pathway functions both alongside and partly independently of canonical ABA signaling.

As climate change is accelerating soil salinization worldwide (Rengasamy, 2010), understanding such early stress‐response mechanisms is vital for guiding the development of salt‐tolerant crops.

## MATERIALS AND METHODS

### Plant materials, growth and stress assays

Arabidopsis (*Arabidopsis thaliana*) Col‐0 plants were used in this research. The following mutants were obtained from the Arabidopsis Stock Center in Columbus, OH: *hkt1* (CS68521), *hkt1‐4* (CS68092), *pp2c49‐1* (SALK_111549C), *pp2c49‐2* (SALK_015078C), *pp2cg1‐1* (SALK_036544C), *pp2cg1‐2* (SALK_200850C). The double mutants *camta6 pp2c49* and camta6 hkt1 were generated through several cycles of genetic crosses between *camta6‐4*, *pp2c49‐1*, and *hkt1*, respectively. Plants expressing *HKT1;1pro*:GUS, *CAMTA6pro*:GUS, and *PP2C49pro*:GUS were created as described previously (Chandran et al., 2024; Shkolnik et al., 2019).

Seed‐surface sterilization and germination assays in the presence of different chemicals were performed as previously described (Shkolnik & Bar‐Zvi, 2008). Germination assays on NaCl, CaCl_2_, sanguinarine (sanguinarine chloride hydrate; Sigma‐Aldrich), or their combination were performed in the same manner. Germination was scored upon the observation of fully unfurled green cotyledons. Treatments with NaCl prior to GUS staining (see GUS staining section further on) and RT‐qPCR were performed by seed imbibition at 4°C in the dark for 2–3 days prior to plating on Whatman filter paper (grade 1) soaked with 0.25X MS medium (Murashige & Skoog, 1962) and supplemented with the indicated chemicals. Germinating embryos (mature embryos) were gently ejected from the seed coat manually, using curved fine forceps, 16 h after starting the treatment. Separation of cotyledon and radicle tissues for RNA isolation and subsequent RT‐qPCR analysis was performed by relatively rough embryo ejection from the seed coat and tissue collection in separate test tubes using a fine pipette under a binocular. Treatment of several‐day‐old seedlings with NaCl, CaCl_2_, sanguinarine, or the indicated combinations was performed by placing the seedlings such that the root was in contact with the agar‐solidified 0.25X MS medium supplemented with the indicated chemicals and the shoot was in the air to avoid direct contact with NaCl, as previously described (Shkolnik et al., 2019; Shkolnik‐Inbar & Bar‐Zvi, 2012). The seedlings were grown vertically in growth chambers under a 16‐h light/8‐h dark photoperiod at 20°C.

### GUS staining

GUS staining was performed as previously described (Weigel & Glazebrook, 2002). Briefly, seeds and seedlings were treated with 300 μl of 90% (v/v) acetone for 1 min, washed with 1 mL staining solution (50 mM sodium phosphate buffer pH 7.0, 2 mM ferricyanide, 2 mM ferrocyanide, and 0.2% v/v Triton X‐100) followed by 1 h incubation at 37°C in staining solution supplemented with 2 mM X‐Gluc (5‐bromo‐4‐chloro‐3‐indolyl‐beta‐D‐glucuronic acid, cyclohexylammonium salt). Images were taken using the Nikon SMZ18 stereoscope system equipped with a DS‐Fi3 camera.

### Quantitative GUS assay

GUS activity was quantified spectrophotometrically as described previously (Jefferson, 1987). Briefly, soluble protein preparations were extracted from 100 mg of GUS‐expressing Arabidopsis embryos and seedlings using GUS extraction buffer containing 50 mM NaPO_4_ pH 7.0, 10 mM beta‐mercaptoethanol, 10 mM Na_2_EDTA, 0.1% v/v sodium lauryl sarcosine, and 0.1% Triton X‐100. The reaction was initiated by mixing 50 μl of protein extract in a 0.5 mL total volume of 1 mM of the GUS substrate 4‐nitrophenyl β‐D‐glucuronide (PNPG). The reaction was incubated at 37°C for 9 h, and absorbance was measured at 415 nm using a spectrophotometer.

### RT‐qPCR analysis

Total RNA was isolated from germinating embryos using the ZR Plant RNA MiniPrep Kit (Zymo Research), and total cDNA was synthesized using the High‐Capacity cDNA Reverse Transcription Kit (Thermo Fisher Scientific). The reaction mixture was prepared according to the manufacturer's instructions, with random primers, and supplemented with 1 μg of total RNA. PCR mixtures (10 μl), containing 5 μl Fast SYBR Green Master Mix (Applied Biosystems by Thermo Fisher Scientific), 500 nM reverse and forward primers designed to amplify 80–150 bp of the genes of interest (Table S4), and 20 ng cDNA were subjected to the Rotor‐Gene Q 5‐Plex HRM real‐time PCR system (Qiagen) using the default program. The *PP2A* (AT1G69960) gene was used as an endogenous control. Relative quantification data were analyzed by the Rotor‐Gene Q Pure Detection software V2.3.5 (Qiagen).

### Ion content analysis

Seeds were sown on filter paper saturated with 0.25X MS medium supplemented with NaCl, sanguinarine, or a combination of both. After 16 h of incubation, embryos were isolated from the seed coat for ion extraction. Sample preparation for Na^+^ and K^+^ quantification was performed as previously described (Kalifa et al., 2004; Shkolnik‐Inbar et al., 2013). In each biological replicate, 40 seedlings per line were sampled. Ion concentrations were measured using an Arcos ICP‐OES spectrometer (Spectro/Ametek).

### Transient promoter activation assay

Promoter fragments of approximately 2 kb (*CAMTA6*, *HKT1;1*, and *PP2CG1*) were amplified using gene‐specific primers listed in Table S4 and subcloned individually into the pCAMBIA1391Z vector upstream of the GUS reporter gene, with the *CAMTA6* and *HKT1;1* promoters inserted using the KpnI/SalI restriction sites and the *PP2CG1* promoter inserted using the PstI/BamHI restriction sites. The *CAMTA6* coding sequence fragment was subcloned into the pBTEX vector downstream of the constitutive CaMV 35S promoter using the KpnI/EcoRI restriction sites to generate the 35S:CAMTA6 (CAMTA6‐OE) construct. Each promoter::GUS fusion construct, either alone (control) or co‐infiltrated with the CAMTA6‐OE construct, was introduced into *Nicotiana benthamiana* leaves via Agrobacterium‐mediated infiltration, after which leaf discs were collected from the infiltrated regions and subjected to GUS staining. The empty pCAMBIA1391Z vector served as an additional control. The procedure was repeated at least 10 times independently.

### Protein extraction and phosphatase activity assay

Crude protein extracts were prepared from roots of 4‐day‐old Arabidopsis seedlings (Col‐0 and *pp2c49‐1*). Root tissue was harvested, flash‐frozen in liquid nitrogen, and ground to a fine powder. Total soluble protein was extracted in ice‐cold buffer containing 50 mM Tris–HCl (pH 7.5), 150 mM NaCl, 0.1% (v/v) Triton X‐100, and 1 mM dithiothreitol (DTT), supplemented with a protease inhibitor cocktail (Roche). Homogenates were centrifuged at 14 000×*g* for 15 min at 4°C, and the clarified supernatant was collected. Protein concentration was determined by the Bradford assay using bovine serum albumin as a standard (Bradford, 1976).

Phosphatase activity was assayed essentially as described by Hurley et al. (2010) with minor modifications. Reaction mixtures (100 μl) contained 10 μg of total protein and 10 mM p‐nitrophenyl phosphate (pNPP; Sigma) in assay buffer (50 mM Tris–HCl pH 7.5, 150 mM NaCl, 5 mM MgCl_2_, 0.1% Triton X‐100, and 1 mM DTT), in the presence or absence of 1 μM sanguinarine. Reactions were incubated at 30°C for 30 min and terminated by the addition of 100 μl 0.5 M NaOH. The release of p‐nitrophenol from pNPP dephosphorylation was quantified by measuring absorbance at 405 nm using a microplate reader. To ensure kinetic linearity, spectrophotometric readings were taken every 10 min over a 60‐min period. All reported data were derived from the 30‐min incubation point, which was confirmed to be within the linear range of product formation. Control reactions without protein were used to correct for non‐enzymatic substrate hydrolysis, and background values were subtracted from all samples. Technical replicates were averaged, and activities were normalized to the mean absorbance of Col‐0 controls, which was set to 1.0.

### Phylogenetic analysis

Gene sequences were retrieved from the TAIR10 database (https://www.arabidopsis.org/). Multiple sequence alignment was performed using MAFFT (v7.450) with default parameters. A maximum likelihood phylogenetic tree was constructed using RAxML (v8.2.12; https://cme.h‐its.org/exelixis/web/software/raxml/). The tree was visualized using FigTree (v1.4.4; http://tree.bio.ed.ac.uk/software/figtree/). Transcriptomic datasets were visualized as heatmaps using the Morpheus web‐based platform (https://software.broadinstitute.org/morpheus/), with data represented as log_2_ fold change (log2FC) values.

### Determination of chlorophyll content

Chlorophyll content in seedlings treated with NaCl, sanguinarine, or a combination of both, as well as in control samples, was quantified by extraction in 80% (v/v) aqueous acetone (Porra et al., 1989). For mature pot‐grown plants, measurements were performed non‐destructively using a CCM‐200 plus Chlorophyll Content Meter (Opti‐Sciences). All chlorophyll data were normalized to the Col‐0 control and are presented as relative chlorophyll content.

### Statistical analysis

Data were analyzed using OriginPro 2024 (OriginLab Corporation) and Microsoft Excel 2021 with the Analysis ToolPak add‐in.

## ACCESSION NUMBERS

Accession numbers of the major genes investigated in this research are: *CAMTA6*, AT3G16940; *PP2C49*, AT3G62260; *HKT1;1*, AT4G10310.

## AUTHOR CONTRIBUTIONS

YK and DS designed the experiments and research plan; YK, AEJC, GS, OC, SA‐P, YW, and DS performed the experiments; YK and DS analyzed the data and wrote the article.

## CONFLICT OF INTEREST

The authors declare no competing interests.

## Supporting information


**Figure S1.** Promoter activity of *CAMTA6*, *HKT1;1*, and *PP2C49* in Arabidopsis seedlings treated with NaCl and/or CaCl_2_.
**Figure S2.** Potential CAMTA‐binding elements in the promoters of *HKT1;1*, *PP2C49*, and *PP2CG1*.
**Figure S3.** Transient activation of gene promoters by CAMTA6 in *Nicotiana benthamiana* leaves.
**Figure S4.** Sanguinarine does not affect ABA‐mediated inhibition of Arabidopsis seed germination.
**Figure S5.** ABA inhibits germination in wild type and mutant Arabidopsis seeds.
**Figure S6.** Effects of sanguinarine and NaCl on germination of wild type and *pp2cg1* mutant Arabidopsis seeds.
**Figure S7.** Sanguinarine (Sang.) modulates the expression of *CAMTA6* and *HKT1;1* in germinating Arabidopsis.
**Figure S8.** *CAMTA6*, *HKT1;1*, and *PP2C49* promoter activity in Arabidopsis seedlings treated with NaCl and/or sanguinarine (Sang.).
**Figure S9.** Effect of salt stress and sanguinarine (Sang.) on seedling growth and chlorophyll content of Arabidopsis genotypes.
**Figure S10.** Phosphatase activity in roots of wild type and *pp2c49‐1* Arabidopsis seedlings in the presence or absence of sanguinarine.
**Figure S11.** Phylogenetic classification and RT‐qPCR validation of *PP2C* genes identified in transcriptome datasets.
**Figure S12.** Distinct transcriptional profiles of PP2C group genes in wild type and Mutant Arabidopsis seedlings under salt stress.
**Figure S13.** A proposed mechanistic framework for the CAMTA6–PP2C49 regulatory circuit and its spatial dynamics.

**Table S1.** List of differentially expressed *PP2C* genes identified in comparisons between wild type *Arabidopsis* (Col‐0) and the *camta6‐5* mutant under control and NaCl‐treated conditions, in germinating seedlings. Genes that met the selection criteria of absolute fold change ≥1.25 and *P* < 0.05 were considered differentially expressed. The following pairwise comparisons are shown: (1) *camta6‐5* NaCl versus *camta6‐5* control; (2) Col‐0 NaCl versus Col‐0 control; (3) *camta6‐5* control versus Col‐0 control; and (4) *camta6‐5* NaCl versus Col‐0 NaCl. Data were extracted from Shkolnik et al. (2019).

**Table S2.** List of differentially expressed *PP2C* genes upregulated and downregulated in response to CaCl_2_, NaCl, and their combination, compared to untreated controls, in germinating seedlings of wild type *Arabidopsis* (Col‐0). Genes that met the selection criteria of absolute fold change ≥1.25 and *P* < 0.05 were considered differentially expressed. The following pairwise comparisons are shown: (1) CaCl_2_ versus control; (2) NaCl versus control; (3) NaCl–CaCl_2_ versus control; (4) NaCl versus CaCl_2_; (5) NaCl–CaCl_2_ versus CaCl_2_; and (6) NaCl–CaCl_2_ versus NaCl. Data were extracted from Chandran et al. (2024).

**Table S3.** List of differentially expressed *PP2C* genes shared between two independent datasets (Chandran et al., 2024; Shkolnik et al., 2019). Genes were included if they met the selection criteria of absolute fold change ≥1.25 and *P* < 0.05 in at least one comparison within each dataset. The table presents *PP2C* genes differentially expressed in germinating seedlings of wild type *Arabidopsis* (Col‐0) in response to control, NaCl, CaCl_2_, and combined NaCl‐CaCl_2_ treatments, and in the *camta6‐5* mutant under control and NaCl‐treated conditions.

**Table S4.** List of primers used in this study.

## Data Availability

No new transcriptomic datasets were generated in this study. The transcriptome data analyzed here were previously reported in Shkolnik et al. (2019) and Chandran et al. (2024). All data supporting the findings of this study are included within the article and its Supplementary Information. Additional materials and datasets are available from the corresponding author upon reasonable request.

## References

[tpj71033-bib-0001] Aburai, N. , Yoshida, M. , Ohnishi, M. & Kimura, K. (2010) Sanguinarine as a potent and specific inhibitor of protein phosphatase 2C *in vitro* and induces apoptosis via phosphorylation of p38 in HL60 cells. Bioscience, Biotechnology, and Biochemistry, 74, 548–552. 20208361 10.1271/bbb.90735

[tpj71033-bib-0002] Antoni, R. , Gonzalez‐Guzman, M. , Rodriguez, L. , Rodrigues, A. , Pizzio, G.A. & Rodriguez, P.L. (2012) Selective inhibition of clade a phosphatases type 2C by PYR/PYL/RCAR abscisic acid receptors. Plant Physiology, 158, 970–980. 22198272 10.1104/pp.111.188623 PMC3271782

[tpj71033-bib-0003] Assaha, D.V.M. , Ueda, A. , Saneoka, H. , Al‐Yahyai, R. & Yaish, M.W. (2017) The role of Na^+^ and K^+^ transporters in salt stress adaptation in glycophytes. Frontiers in Physiology, 8, 509. 28769821 10.3389/fphys.2017.00509 PMC5513949

[tpj71033-bib-0004] Berthomieu, P. , Conéjéro, G. , Nublat, A. , Brackenbury, W.J. , Lambert, C. , Savio, C. et al. (2003) Functional analysis of AtHKT1 in Arabidopsis shows that Na^+^ recirculation by the phloem is crucial for salt tolerance. The EMBO Journal, 22, 2004–2014. 12727868 10.1093/emboj/cdg207 PMC156079

[tpj71033-bib-0005] Bradford, M.M. (1976) A rapid and sensitive method for the quantitation of microgram quantities of protein utilizing the principle of protein‐dye binding. Analytical Biochemistry, 72(1), 248–254. 942051 10.1016/0003-2697(76)90527-3

[tpj71033-bib-0006] Byrt, C.S. , Xu, B. , Krishnan, M. , Lightfoot, D.J. , Athman, A. , Jacobs, A.K. et al. (2014) The Na^+^ transporter, TaHKT1;5‐D, limits shoot Na^+^ accumulation in bread wheat. The Plant Journal: For Cell and Molecular Biology, 80, 516–526. 25158883 10.1111/tpj.12651

[tpj71033-bib-0007] Chandran, A.E.J. , Finkler, A. , Hait, T.A. , Kiere, Y. , David, S. , Pasmanik‐Chor, M. et al. (2024) Calcium regulation of the Arabidopsis Na^+^/K^+^ transporter HKT1;1 improves seed germination under salt stress. Plant Physiology, 194, 1834–1852. 38057162 10.1093/plphys/kiad651 PMC10904324

[tpj71033-bib-0008] Chu, M. , Chen, P. , Meng, S. , Xu, P. & Lan, W. (2021) The Arabidopsis phosphatase PP2C49 negatively regulates salt tolerance through inhibition of AtHKT1;1. Journal of Integrative Plant Biology, 63, 528–542. 32877013 10.1111/jipb.13008

[tpj71033-bib-0060] da Costa Silva, O. (1994) CG‐1, a parsley light‐induced DNA‐binding protein. Plant Molecular Biology, 25, 921–924. 8075408 10.1007/BF00028887

[tpj71033-bib-0009] Davenport, R.J. , Muñoz‐Mayor, A. , Jha, D. , Essah, P.A. , Rus, A. & Tester, M. (2007) The Na^+^ transporter AtHKT1;1 controls retrieval of Na^+^ from the xylem in Arabidopsis. Plant, Cell & Environment, 30, 497–507. 10.1111/j.1365-3040.2007.01637.x 17324235

[tpj71033-bib-0010] Deinlein, U. , Stephan, A.B. , Horie, T. , Luo, W. , Xu, G. & Schroeder, J.I. (2014) Plant salt‐tolerance mechanisms. Trends in Plant Science, 19, 371–379. 24630845 10.1016/j.tplants.2014.02.001 PMC4041829

[tpj71033-bib-0011] Dodd, A.N. , Kudla, J. & Sanders, D. (2010) The language of calcium signaling. Annual Review of Plant Biology, 61, 593–620. 10.1146/annurev-arplant-070109-104628 20192754

[tpj71033-bib-0012] Doherty, C.J. , van Buskirk, H.A. , Myers, S.J. & Thomashow, M.F. (2009) Roles for Arabidopsis CAMTA transcription factors in cold‐regulated gene expression and freezing tolerance. The Plant Cell, 21, 972–984. 19270186 10.1105/tpc.108.063958 PMC2671710

[tpj71033-bib-0013] Finkler, A. , Ashery‐Padan, R. & Fromm, H. (2007) CAMTAs: calmodulin‐binding transcription activators from plants to human. FEBS Letters, 581, 3893–3898. 17689537 10.1016/j.febslet.2007.07.051

[tpj71033-bib-0014] Finkler, A. , Kaplan, B. & Fromm, H. (2007) Ca^2+^‐responsive cis‐elements in plants. Plant Signaling & Behavior, 2, 17–19. 19704800 10.4161/psb.2.1.3611 PMC2633890

[tpj71033-bib-0015] Fujii, H. , Chinnusamy, V. , Rodrigues, A. , Rubio, S. , Antoni, R. , Park, S.Y. et al. (2009) In vitro reconstitution of an abscisic acid signalling pathway. Nature, 462, 660–664. 19924127 10.1038/nature08599 PMC2803041

[tpj71033-bib-0016] Galon, Y. , Finkler, A. & Fromm, H. (2010) Calcium‐regulated transcription in plants. Molecular Plant, 3, 653–669. 20457642 10.1093/mp/ssq019

[tpj71033-bib-0017] Hassani, A. , Azapagic, A. & Shokri, N. (2021) Global predictions of primary soil salinization under changing climate in the 21^st^ century. Nature Communications, 12, 1–17. 10.1038/s41467-021-26907-3 PMC8602669 34795219

[tpj71033-bib-0018] Houser, F. & Horie, T. (2010) A conserved primary salt tolerance mechanism mediated by HKT transporters: a mechanism for sodium exclusion and maintenance of high K^+^/Na^+^ ratio in leaves during salinity stress. Plant, Cell & Environment, 33, 552–565. 10.1111/j.1365-3040.2009.02056.x 19895406

[tpj71033-bib-0019] Horie, T. , Horie, R. , Chan, W.Y. , Leung, H.‐Y. & Schroeder, J.I. (2006) Calcium regulation of sodium hypersensitivities of *sos3* and *athkt1* mutants. Plant & Cell Physiology, 47, 622–633. 16540484 10.1093/pcp/pcj029

[tpj71033-bib-0020] Hu, W. , Yan, Y. , Hou, X. , He, Y. , Wei, Y. , Yang, G. et al. (2015) TaPP2C1, a group F2 protein phosphatase 2C gene, confers resistance to salt stress in transgenic tobacco. PLoS One, 10, e0129589. Available from: 10.1371/journal.pone.0129589 26057628 PMC4461296

[tpj71033-bib-0021] Hurley, B.A. , Tran, H.T. , Marty, N.J. , Park, J. , Snedden, W.A. , Mullen, R.T. et al. (2010) The dual‐targeted purple acid phosphatase isozyme AtPAP26 is essential for efficient acclimation of Arabidopsis to nutritional phosphate deprivation. Plant Physiology, 153, 1112–1122. 20348213 10.1104/pp.110.153270 PMC2899917

[tpj71033-bib-0022] Jefferson, R.A. (1987) Assaying chimeric genes in plants: the GUS gene fusion system. Plant Molecular Biology Reporter, 5, 387–405.

[tpj71033-bib-0023] Kalifa, Y. , Perlson, E. , Gilad, A. , Konrad, Z. , Scolnik, P.A. & Bar‐Zvi, D. (2004) Over‐expression of the water and salt stress‐regulated *Asr1* gene confers an increased salt tolerance. Plant, Cell & Environment, 27, 1459–1468.

[tpj71033-bib-0024] Kaplan, B. , Davydov, O. , Knight, H. , Galon, Y. , Knight, M.R. , Fluhr, R. et al. (2006) Rapid transcriptome changes induced by cytosolic Ca^2+^ transients reveal ABRE‐related sequences as Ca^2+^‐responsive cis elements in Arabidopsis. The Plant Cell Online, 18, 2733–2748. 10.1105/tpc.106.042713 PMC1626612 16980540

[tpj71033-bib-0025] Kim, Y.S. , An, C. , Park, S. , Gilmour, S.J. , Wang, L. , Renna, L. et al. (2017) CAMTA‐mediated regulation of salicylic acid immunity pathway genes in Arabidopsis exposed to low temperature and pathogen infection. Plant Cell, 29, 2465–2477. 28982964 10.1105/tpc.16.00865 PMC5774559

[tpj71033-bib-0026] Liu, X. , Zhu, Y. , Zhai, H. , Cai, H. , Ji, W. , Luo, X. et al. (2012) AtPP2CG1, a protein phosphatase 2C, positively regulates salt tolerance of Arabidopsis in abscisic acid‐dependent manner. Biochemical and Biophysical Research Communications, 422, 710–715. 22627139 10.1016/j.bbrc.2012.05.064

[tpj71033-bib-0027] Mäser, P. , Eckelman, B. , Vaidyanathan, R. , Horie, T. , Fairbairn, D.J. , Kubo, M. et al. (2002) Altered shoot/root Na^+^ distribution and bifurcating salt sensitivity in Arabidopsis by genetic disruption of the Na^+^ transporter AtHKT1. FEBS Letters, 531, 157–161. 12417304 10.1016/s0014-5793(02)03488-9

[tpj71033-bib-0028] Merlot, S. , Gosti, F. , Guerrier, D. , Vavasseur, A. & Giraudat, J. (2001) The ABI1 and ABI2 protein phosphatases 2C act in a negative feedback regulatory loop of the abscisic acid signalling pathway. The Plant Journal, 25, 295–303. 11208021 10.1046/j.1365-313x.2001.00965.x

[tpj71033-bib-0029] Møller, I.S. , Gilliham, M. , Jha, D. , Mayo, G.M. , Roy, S.J. , Coates, J.C. et al. (2009) Shoot Na^+^ exclusion and increased salinity tolerance engineered by cell type‐specific alteration of Na^+^ transport in Arabidopsis. The Plant Cell, 21, 2163–2178. 19584143 10.1105/tpc.108.064568 PMC2729596

[tpj71033-bib-0030] Munns, R. & Tester, M. (2008) Mechanisms of salinity tolerance. Annual Review of Plant Biology, 59, 651–681. 10.1146/annurev.arplant.59.032607.092911 18444910

[tpj71033-bib-0031] Murashige, T. & Skoog, F. (1962) A revised medium for rapid growth and bio assays with tobacco tissue cultures. Physiologia Plantarum, 15, 473–497.

[tpj71033-bib-0032] Nonogaki, H. , Bassel, G.W. & Bewley, J.D. (2010) Germination‐still a mystery. Plant Science, 179, 574–581.

[tpj71033-bib-0033] Pandey, G.K. , Yong, H.C. , Kim, K.N. , Grant, J.J. , Li, L. , Hung, W. et al. (2004) The calcium sensor calcineurin B‐like 9 modulates abscisic acid sensitivity and biosynthesis in Arabidopsis. The Plant Cell, 16, 1912–1924. 15208400 10.1105/tpc.021311 PMC514170

[tpj71033-bib-0034] Park, S.Y. , Fung, P. , Nishimura, N. , Jensen, D.R. , Fujii, H. , Zhao, Y. et al. (2009) Abscisic acid inhibits type 2C protein phosphatases via the PYR/PYL family of START proteins. Science, 324, 1068–1071. 19407142 10.1126/science.1173041 PMC2827199

[tpj71033-bib-0035] Peharec‐Štefanić, P. , Koffler, T. , Adler, G. & Bar‐Zvi, D. (2013) Chloroplasts of salt‐grown Arabidopsis seedlings are impaired in structure, genome copy number and transcript levels. PLoS One, 8, e82548. 24340039 10.1371/journal.pone.0082548 PMC3855474

[tpj71033-bib-0036] Porra, R.J. , Thompson, W.A. & Kriedemann, P.E. (1989) Determination of accurate extinction coefficients and simultaneous equations for assaying chlorophylls a and b extracted with four different solvents: verification of the concentration of chlorophyll standards by atomic absorption spectroscopy. Biochimica et Biophysica Acta, 975, 384–394.

[tpj71033-bib-0037] Rahman, H. , Yang, J. , Xu, Y.P. , Munyampundu, J.P. & Cai, X.Z. (2016) Phylogeny of plant CAMTAs and role of AtCAMTAs in nonhost resistance to *Xanthomonas oryzae* pv. *Oryzae* . Frontiers in Plant Science, 7, 1–17. 26973658 10.3389/fpls.2016.00177 PMC4770041

[tpj71033-bib-0038] Rengasamy, P. (2010) Soil processes affecting crop production in salt‐affected soils. Functional Plant Biology, 37, 613–620.

[tpj71033-bib-0039] Saez, A. , Apostolova, N. , Gonzalez‐Guzman, M. , Gonzalez‐Garcia, M.P. , Nicolas, C. , Lorenzo, O. et al. (2004) Gain‐of‐function and loss‐of‐function phenotypes of the protein phosphatase 2C HAB1 reveal its role as a negative regulator of abscisic acid signalling. The Plant Journal, 37, 354–369. 14731256 10.1046/j.1365-313x.2003.01966.x

[tpj71033-bib-0040] Schweighofer, A. , Hirt, H. & Meskiene, I. (2004) Plant PP2C phosphatases: emerging functions in stress signaling. Trends in Plant Science, 9, 236–243. 15130549 10.1016/j.tplants.2004.03.007

[tpj71033-bib-0061] Shkolnik, D. , & Bar‐Zvi, D. (2008) Tomato ASR1 abrogates the response to abscisic acid and glucose in Arabidopsis by competing with ABI4 for DNA binding. Plant Biotechnology Journal, 6(4), 368–378. 18363631 10.1111/j.1467-7652.2008.00328.x

[tpj71033-bib-0041] Shkolnik, D. , Finkler, A. , Pasmanik‐Chor, M. & Fromm, H. (2019) Calmodulin‐binding transcription activator 6: a key regulator of Na^+^ homeostasis during germination. Plant Physiology, 180, 1101–1118. 30894419 10.1104/pp.19.00119 PMC6548231

[tpj71033-bib-0042] Shkolnik‐Inbar, D. , Adler, G. & Bar‐Zvi, D. (2013) ABI4 downregulates expression of the sodium transporter HKT1;1 in Arabidopsis roots and affects salt tolerance. The Plant Journal, 73, 993–1005. 23240817 10.1111/tpj.12091

[tpj71033-bib-0043] Shkolnik‐Inbar, D. & Bar‐Zvi, D. (2012) A simple physiologically relevant double‐agar‐layer method for post‐germination treatment of seedlings. Plant Growth Regulation, 67, 305–310.

[tpj71033-bib-0044] Steinhorst, L. & Kudla, J. (2013) Calcium‐a central regulator of pollen germination and tube growth. BBA‐Molecular Cell Research, 1833, 1573–1581. 23072967 10.1016/j.bbamcr.2012.10.009

[tpj71033-bib-0045] Sun, Y. , Kong, X. , Li, C. , Liu, Y. & Ding, Z. (2015) Potassium retention under salt stress is associated with natural variation in salinity tolerance among Arabidopsis accessions. PLoS One, 10, 4–8. 10.1371/journal.pone.0124032 PMC4438003 25993093

[tpj71033-bib-0046] Sunarpi, H.T. , Horie, T. , Motoda, J. , Kubo, M. , Yang, H. , Yoda, K. et al. (2005) Enhanced salt tolerance mediated by AtHKT1 transporter‐induced Na^+^ unloading from xylem vessels to xylem parenchyma cells. The Plant Journal, 44, 928–938. 16359386 10.1111/j.1365-313X.2005.02595.x

[tpj71033-bib-0047] Turan, S. & Tripathy, B.C. (2015) Salt‐stress induced modulation of chlorophyll biosynthesis during de‐etiolation of rice seedlings. Physiologia Plantarum, 153, 477–491. 25132047 10.1111/ppl.12250

[tpj71033-bib-0048] Umezawa, T. , Sugiyama, N. , Mizoguchi, M. , Hayashi, S. , Myouga, F. , Yamaguchi‐Shinozaki, K. et al. (2009) Type 2C protein phosphatases directly regulate abscisic acid‐activated protein kinases in Arabidopsis. Proceedings of the National Academy of Sciences of the United States of America, 106, 17588–17593. 19805022 10.1073/pnas.0907095106 PMC2754379

[tpj71033-bib-0049] Uozumi, N. , Kim, E.J. , Rubio, F. , Yamaguchi, T. , Muto, S. , Tsuboi, A. et al. (2000) The Arabidopsis HKT1 gene homolog mediates inward Na currents in Xenopus laevis oocytes and Na uptake in Saccharomyces cerevisiae 1. Plant Physiology, 122, 1249–1259. 10759522 10.1104/pp.122.4.1249 PMC58961

[tpj71033-bib-0050] Vogt, A. , Tamewitz, A. , Skoko, J. , Sikorski, R.P. , Giuliano, K.A. & Lazo, J.S. (2005) The benzo[c]phenanthridine alkaloid, sanguinarine, is a selective, cell‐active inhibitor of mitogen‐activated protein kinase phosphatase‐1. Journal of Biological Chemistry, 280, 19078–19086. 15753082 10.1074/jbc.M501467200

[tpj71033-bib-0051] Wang, M. , Zheng, Q. , Shen, Q. & Guo, S. (2013) The critical role of potassium in plant stress response. International Journal of Molecular Sciences, 14, 7370–7390. 23549270 10.3390/ijms14047370 PMC3645691

[tpj71033-bib-0052] Wang, W. , Vinocur, B. & Altman, A. (2003) Plant responses to drought, salinity and extreme temperatures: towards genetic engineering for stress tolerance. Planta, 218, 1–14. 14513379 10.1007/s00425-003-1105-5

[tpj71033-bib-0053] Wang, Y. & Wu, W.H. (2013) Potassium transport and signaling in higher plants. Annual Review of Plant Biology, 64, 451–476. 10.1146/annurev-arplant-050312-120153 23330792

[tpj71033-bib-0054] Weigel, D. & Glazebrook, J. (2002) Arabidopsis. A laboratory manual. Cold Spring Harbor, NY: Cold Spring Harbor Laboratory Press.

[tpj71033-bib-0055] Whalley, H.J. & Knight, M.R. (2013) Calcium signatures are decoded by plants to give specific gene responses. The New Phytologist, 197, 690–693. 23190495 10.1111/nph.12087

[tpj71033-bib-0056] Xue, T. , Wang, D. , Zhang, S. , Ehlting, J. , Ni, F. , Jakab, S. et al. (2008) Genome‐wide and expression analysis of protein phosphatase 2C in rice and Arabidopsis. BMC Genomics, 9, 1–21. 19021904 10.1186/1471-2164-9-550 PMC2612031

[tpj71033-bib-0057] Yang, T. , Peng, H. , Whitaker, B.D. & Conway, W.S. (2012) Characterization of a calcium/calmodulin‐regulated SR/CAMTA gene family during tomato fruit development and ripening. BMC Plant Biology, 12, 19. 22330838 10.1186/1471-2229-12-19 PMC3292969

[tpj71033-bib-0058] Yip Delormel, T. & Boudsocq, M. (2019) Properties and functions of calcium‐dependent protein kinases and their relatives in *Arabidopsis thaliana* . New Phytologist, 224, 585–604. 31369160 10.1111/nph.16088

[tpj71033-bib-0059] Zhu, J.K. (2002) Salt and drought stress signal transduction in plants. Annual Review of Plant Biology, 53, 247–273. 10.1146/annurev.arplant.53.091401.143329 PMC3128348 12221975

