# Supplementary material for: The Ca2+‐CAMTA6‐PP2C49‐HKT1;1 signaling axis modulates ion homeostasis during Arabidopsis seed germination under salt stress
Source: Plant J. 2026 Jul 9;127(1):e71033. doi: 10.1111/tpj.71033 (PMC13349281; doi:10.1111/tpj.71033)
Supplement: Supplementary file 1 — Figure S1. Promoter activity of CAMTA6, HKT1;1, and PP2C49 in Arabidopsis seedlings treated with NaCl and/or CaCl2. Figure S2. Potential CAMTA‐binding elements in the promoters of HKT1;1, PP2C49, and PP2CG1. Figure S3. Transient activation of gene promoters by CAMTA6 in Nicotiana benthamiana leaves. Figure S4. Sanguinarine does not affect ABA‐mediated inhibition of Arabidopsis seed germination. Figure S5. ABA inhibits germination in wild type and mutant Arabidopsis seeds. Figure S6. Effects of sanguinarine and NaCl on germination of wild type and pp2cg1 mutant Arabidopsis seeds. Figure S7. Sanguinarine (Sang.) modulates the expression of CAMTA6 and HKT1;1 in germinating Arabidopsis. Figure S8. CAMTA6, HKT1;1, and PP2C49 promoter activity in Arabidopsis seedlings treated with NaCl and/or sanguinarine (Sang.). Figure S9. Effect of salt stress and sanguinarine (Sang.) on seedling growth and chlorophyll content of Arabidopsis genotypes. Figure S10. Phosphatase activity in roots of wild type and pp2c49‐1 Arabidopsis seedlings in the presence or absence of sanguinarine. Figure S11. Phylogenetic classification and RT‐qPCR validation of PP2C genes identified in transcriptome datasets. Figure S12. Distinct transcriptional profiles of PP2C group genes in wild type and Mutant Arabidopsis seedlings under salt stress. Figure S13. A proposed mechanistic framework for the CAMTA6–PP2C49 regulatory circuit and its spatial dynamics. [file TPJ-127-0-s003.docx]

Supplementary material for:

**The Ca²⁺-CAMTA6-PP2C49-HKT1;1 signaling axis modulates ion homeostasis during Arabidopsis seed germination under salt stress**

Yvonne Kiere, Ancy EJ Chandran, Guy Sobol^†^, Omer Cremer, Shaked Azoulay-Portal, Yonatan Wexler, Doron Shkolnik^*^

Robert H. Smith Institute of Plant Sciences and Genetics in Agriculture, Faculty of Agriculture, Food and Environment, Hebrew University of Jerusalem, Rehovot 7610001, Israel

^†^Present address: Department of Bacteriology, University of Wisconsin–Madison, Madison, WI 53706, USA

*Author for correspondence: [doron.shkolnik@mail.huji.ac.il](mailto:doron.shkolnik@mail.huji.ac.il)

**List of contents**

1. Supplementary Figures S1–S13.
2. Supplementary Tables S1-S3 (legends) and S4 (primer list).


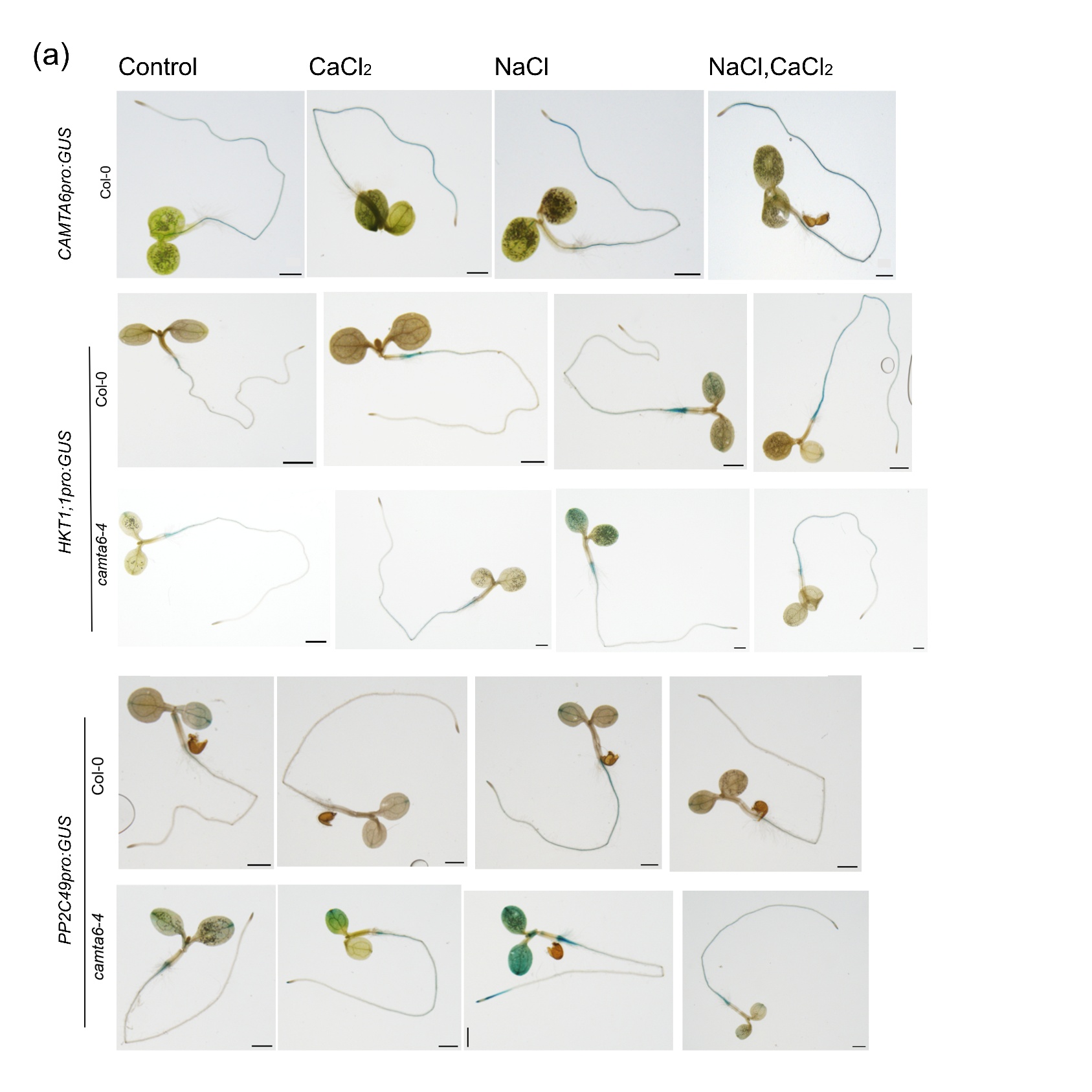


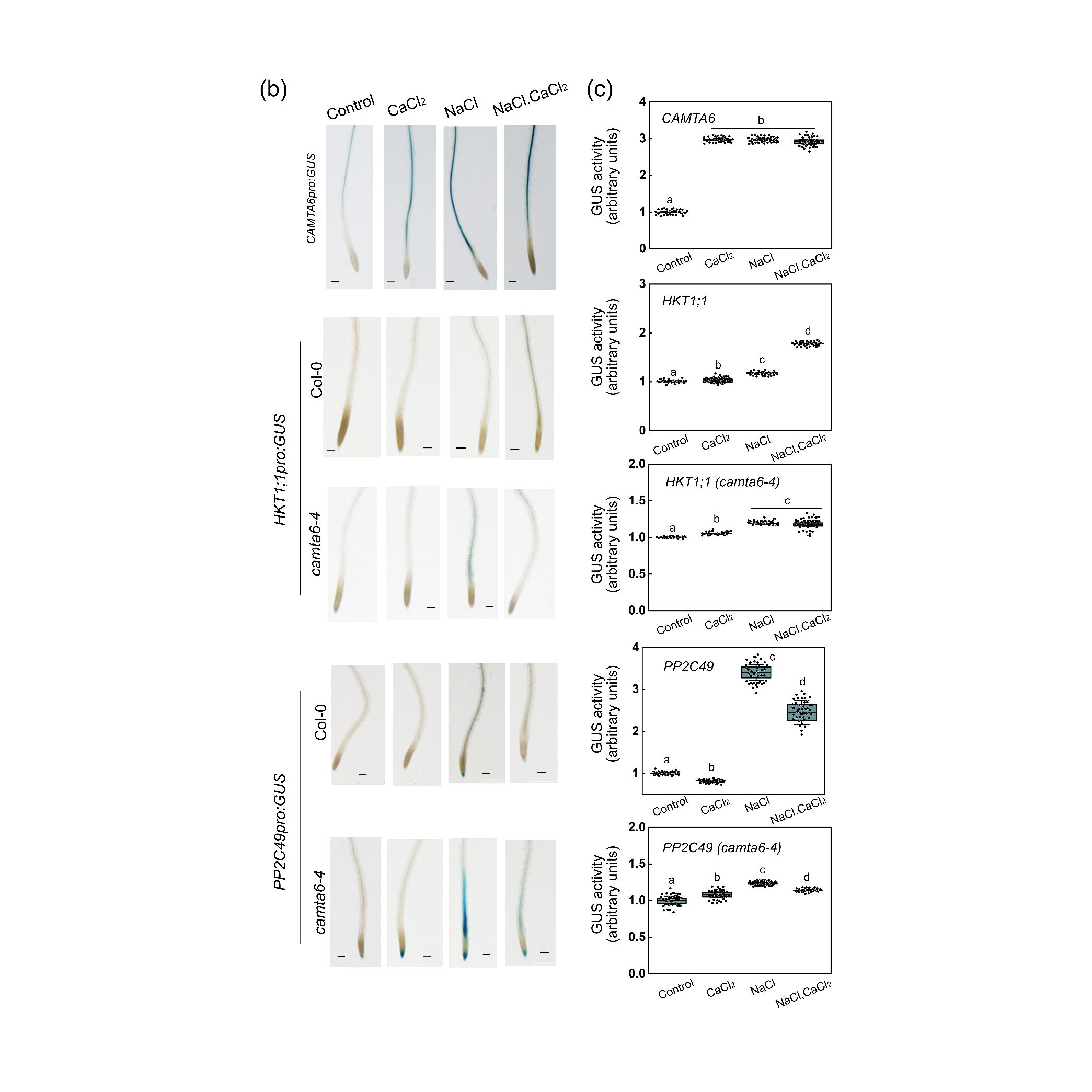


**Figure S1**. Promoter activity of *CAMTA6*, *HKT1;1*, and *PP2C49* in Arabidopsis seedlings treated with NaCl and/or CaCl₂.

(a, b) Five-day-old seedlings of the indicated genotypes were treated with the specified chemicals (NaCl, 150 mM; CaCl_2_, 10 mM), GUS-stained (see Materials and Methods), and imaged using a stereomicroscope system. Whole seedlings are shown in (a) and young primary root zones in (b) (bars = 250 µm for a; 100 µm for b). (c) Spectrophotometric GUS assay. Relative GUS activity was quantified using PNPG as a substrate as described previously (Jefferson, 1987). Bold lines in each box indicate the mean, and whiskers represent ± SE (three independent biological experiments, n = 45). Top and bottom sides of the boxes correspond to the third and first quartiles, respectively. Different lowercase letters indicate significantly different values by Tukey’s HSD post hoc test (*P* < 0.005). Individual data points for each replicate are shown as dots overlaid on the box plots.

***HKT1;1* (AT4G10310) promoter**

TGGTCATCTATATTTCTCAAATAGTTTAATGTGGGTGCTCTTAGAGTATGGTCATTTATATTTTCTCAAATAGTTTTATTTTGTCAACGTTTGGTGTATCAACTATTTTGCAAATAGCAGATACTTTCAGTGAAATTCCTTATCATCGAGGTCATACCTCCTTTGGCATACATTGTAAAGTCTCTAACATAAGCCAACAACTTGCAAGCTCTAATGATTCACAAGTTGATAACACCATTTTGCAAGCTCTAATAATGATTCATTAAACAAATTGGCAATTTTCAAATACCAACACCACCCTTTTTCTTGTTAAAATTACTATTGTTTATTTTATTTTATTTAATGTTAAATTATATTTAAAAGAAAAATAACCTTTACATATGAAATGCTAACTTTTTCAAGAGTTATTTTAAAAAAACAGAATTTCTAATATATCTATTGTCTTGATTCAAACCAAATTTGGATGCCATTTTTGAACTTCAATCCTCCACCACCTTGAATTGTGCTCAAACGGTTTCTAATATTCTTGTCTATGATCATCACAAGTATTTCCGTTGGTGATGATTGCTCCCCATGCCTCCTCCTATTCTGTTCATTCCATATTGAGTGTATAGAGTTCTGTAGTGCATACCGAAGTATGTAGCTCTTCGTTTTGTCCACAAGGTTTTCAGATAAAATCTTCATAACCTCTGTCCAACTCACCGAGAATCTGTCCGAGAAAATTCCCCTTGTTAGGTCTTCCCATATCTTTTTGAGTATAGGCAGTGGAAAAATAAGTGTTCCCTCGTCTCTACTCGTTCATTGCAAAAAATGCAGCTCGAATCTACATTACAATTCCACTTCCTCATTCTCTCCCCTGTTGCGACTCTGTTTTTTACAACCGTATTGTTATACTTTGGAGTAGCGTATGGGAACCATATCTCATTTTGTCCCTCCATCGGCGGCTTTGCTAACCGCAGCTGTGACCAAGAACATTTATTTTTCCCTTTTAACAGAACTATATCGTGATCGTTCAGTTCTCCATTCAGCTTCTGTTTTCTTATTTCTTCTTCAACACGGTTTAAGATACCCTTTCGATGGTTTCTCCTTCGTGTCATGGACATCACCTCTTTCATTGTGCTTGAACTAGGAATCCCTAGATCAATGCATCCTCTCTAAGACATCCCATGTTACTCCATGTGTCAATACCAAAAAGACGTAGTTTCGCCGCTTTTAACCACTACCATGACTGTTGATCTCTTTAAATAATTAAAAAGTGGTGTTCCCCATGAAGACCGTTCACTTCCATCAATTTATGGTCTTCTTGGACCAATTTCGTTTATCCAAACTATTACCCCTACAACATCGTTTATAAATTGTCTTTACATTTTGATCTAGAATTTGATCTATTTTGCACGAATCCTCTTCCACCTTTTCAGGCATGTACGAGCAATATAACATGAGTTTGATCAAATTTTGCATGGTCTCTACGAAGTTGAGTCATGCAATTTTTACCTATAATTTTTGTTCTCGGCATTTTTATAGCTCTCTACTTAGGATATAAGTAACATGGAGTTGGATGAATCCTTTTCTTTATATTGAAATCGAATCTTCAATGCTAAAAATAAAATCCACTCAATCAGTGAGAGAATTATTATGGAACAAGCTATAAAGACGAAAGAATCACTCAATCATACAATAAAGAAGGATGTTTTTTTCATTTAAACTAAGAAAGAGAACTTTTCCACGTGAATTAAAATAGACATCTCAATAATAAAGACGATTTTTTTTCTTCTCTTTTTCCTCTCACTTTGTATTGTATTGTATTTCTATATATTTCTTCCTCTTACAAATACCCTTTTAAATTGGAAAGAAAAAACAGGAATCGCTATCATCAGTAATAGTCATCATTAATCAATTTATATGTAATATGTGGCTGACAATTTCCATGTACGTGTAATATGTAATATATAAACACAACTTATGGCCAGTATAATATTAATGCTTAAACCGACTCGAGAACTAAA

***PP2C49* (AT3G62260) promoter**

TTAGTGGCTTTTGCCTTTTGGTTATGTGGTTGTCTTAAACTGTGTTTGTTGCGTCTCGATTTTTATTGTCGGATCAGAGATTAGATTAAGACATTCTCATTGACCATTTTTCCACGCGTAGGTACAGTAACTTAACCTGATTAAAACCATTCTAGTTTGACTAATATAATGTTTTAAAGATCCTTCGCAGAATTTAAATACAAAAAAGGGCTAAAAGTTGCACCTTTTTCTTATTAATATTATAGTCGAAATCTTAAGGCCAACTATTCCCTTAAAATCCAACAAGGAAGAAAACCTTACTGAATCCAATGGGCTTAATACTTTCAGACAGTTTAAAGATTTATGGCTTTCCTCGAAATTATGATCCATTAAATGCGTGTCTCCTCATTGATGATCATCAGCAAATCGTGTGTCATATTATATTAACCCTAATCATAATATATTAATACTATAACACCAGCAACATTATGCTGAGTCATACATATATATGCTAATAAGTACTAGTAGTTGTTAATTTTTATTGGTAAGATACCATAACACTAGTTTTATATTAGACTTTACACGAACAAGTTTCAAAATTTTAGCAACGCATTAGATCTTGAGCATCTTGGTTTCGTCTAAAGAAACATTATTTAGTTGAATGTCTTACATAGTAAGACAAATATTTTTGGGTAATATATAAAAAATGTGATAATTTTTAACTTTTAAATTAGTATTTAGGTTAAATTAGATCATACATTCTTAATATGATAACAAAGTGTATTACCGCCTTAATAGACAGTTTTTTTATAAAGAATGGTTTTGCACCTACTCTATACAAATAACATGTCTTATTATATTTTCGAAATGTTAAACTTACTCATATTGCTTAGGACAAAATTTTTAAGTAATAAATAAAACGTGTGGTAATTCTTAAATTTGGATTACTATGTCAGTTTAGATATTTTAATTAATTTTTCCAATAGTCAAATTGAGAAGGAAAAAACAACCACATAAAATAATAAATTAGGTAATGGCTTTGGATCATAGAGAGCACTTAACTTGTTGATCTACGCAATGTGTAAAGCTTGATACCACTAAACTATTATTCGTGACAAAAATTAAAAAGAAAGAAAAGACTGCTGACCTGGCATGACAGATAGTTAAACCTTGGGAGGCAAGTTCATCCTGGTGATTTTAACGGTCCAGATTCATCTAGATGCAACGCCATTGTGTGATTAAAGATCGTCACTTGCAGGTGTGTACACGGACAAACCTTTAGAGATTCTGGTCGAGAAAGTTCTGTACTTCCTAAAGTACCCCTAAACATTTAATCCTATTACAGAAATATCCCCGTTATTTCCAAAGAAAGTCCTCGGAAGTTGGTAAAAGTTTAAAAAGGAGTGGTTCAGAATCCGACGTCGTTCGAGGGTACGCGCGTCAAAGCTCGAATGACACGTGTCATAATTAGGGCTTAGTCTTAGCCGTGTGTAATCCACAAACTAAACAGAGATCATAATCTACATAAGCAACCGACTCGCCACAGATGATGGCCACGTGTCAACTGAATTTTACCGGTTACCCAGTCACCCATAATCGAGTCTTTTTCTTCTTATATATAATCAGAGAGAAAAAGCCCACGCGGCTCAGAAATTTCGTCCACGAGAGGAATAAATTCACACGCGAACTTCGATAGTTGTTAATTTTCGTTGAACACAAGTGACGAATTTGAGAGAGAAAAAAAAAGAAAATCAGAGATAAAGAATTTGAGTTTCTTGGAATTAAAGGATCGATTTTGTTGAAGTGAAATTGAAATTGATTGTGAAAAGAGAAAGAGACTG

***PP2CG1* (AT2G33700) promoter**

ATTATACGCCAAATTTGTAAAAATATTTGGTCAAGAAAACTCTCGTACGTGTCATTTCATGACATATATTGTAGCACAAGTTGTGGTGACCTACTGATTGTCTGATCGATAGCATATTAGAAAAGAAATAAAATGAACCTTAAAAGTCAAATCCTTAACAACTACTAGCAATTAACAAACATGTTTGAATAGAAGTTTCAAGAATTCACATTGAAAACTTTACTCTCTCTTTTCAAACATGTTTCATCAAACGGAAATGTAATATTTAGTCCACTAAATCTCTAATTAAAGTCAGTAATTGATAGTTACAAAATTAATATGCTTCTTCCATCGGTATGAATTAATTATGATGCCCAACAGGCAACAACTAGCTACCAATTTGATTTCTACCGGCTTTGATCAACCTAAACCGGAAATAAATTTCTCGGTAATGTGAACACAGCCGCAACATTAGCTGAAACCAATATAATTACGACACGTGTATGGCGGGAATCAGACGCCGCAACTTTAATTTTTTAATCGGAGGTAACCCCTCGCCATTGGGAAAGACAAAAGGAGATTTGCATGGTAGCGATCTTGACCGTCGATGTTCTATCTTAGAGATCTAATGGTTAGGAATTTATGGCGGTACCACCCGGTCAAAGTAATGCGTACCGTCGATATTTGACACGTGTCGGTAATTTAGCTCCGGTTTGACAGATAGACGATACGAGAGATTACCTGGAAACGCCGGTGGCCAACCAAATACCGAACGAGACAAGCTTCTCTCTCTCTTTCTCTATCTATCTTTCTCTCTCAAAGAAAGCTTTTGGAAATTGTCTTTCGTCAAATTCTGTTCTTCTTCTTCATTAAACTCATAACTTTTTCTGGGTTTTTCGAGAATTTCTTCGAAAGGATCTTACGGTAAGAGAAAAGGAAAATTAAGAGGGAATAAGAAGATTCATCATCATTGTCATCTGTGGGGAATCTCAGAGCTTTTTTTTCTGTAGTTTTGTTATCTTTCTGAATTCTCTACCTGCTTCAAGATTTCATTTTTATTCATCGATCTTTGGGGTTTAGTTTCAAAGCTGAATTCTTTGAGGTTTCTGAAATTTGCGAGCTGGGTTTTTGTTGAAATAGGCTTCTGAGTTTTGGTTATATTTGTGGATAATCGATTACTTGATCTGACCTGAGAAAAAGTAGAAACCTTTGAGTAGAGCTGGTTCTATTACAAAGTTGTGTTTGGGAGCTTGCTACAAGACCATTGGATTTCTTCACTTTCTTCATTTGTTGAGTCTGCTACAAGATCAAGGATCTGTTGATTTTGGGTATTTGATCAAAATCTGAAATTGATCTTGTGGAAGTAAGGTTGCTGTAGTTGTCAAGGTCTTTGGTGAAGTTTGTTTCTCACAAAGTTTGATCCTTGAAGTTGGTCTCTATTGATCTTAGATCTAGTGTGAATAAAGGAAGTCCTTGGAAAGCAGCAGACTTGAAAACGCTATCACAAGAGGAATTGCTTCCTGAAGAGACTGTCTGGATTGCTGATAGATTTATATGATTCAGAGAAGAACTCACCG

**Figure S2**. Potential CAMTA-binding elements in the promoters of *HKT1;1*, *PP2C49*, and *PP2CG1*.

Promoter sequences were obtained from the TAIR database (Arabidopsis.org). The ABRE/CAMTA-binding element (CACGTGTC) and its coupling element ([C/A]ACGCG[T/C/G]) are highlighted.


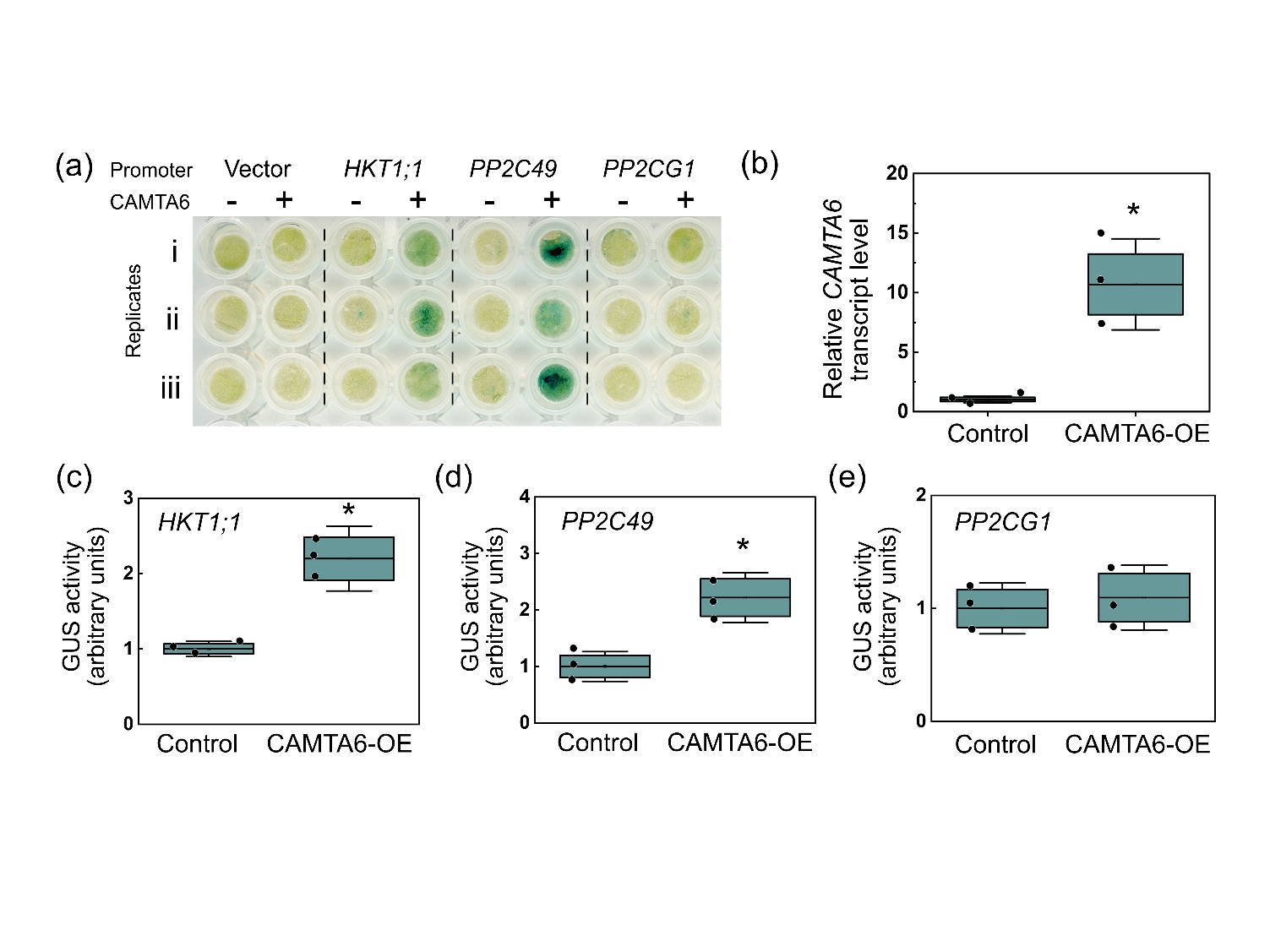


**Figure S3**. Transient activation of gene promoters by CAMTA6 in *Nicotiana benthamiana* leaves.

(a) Gene promoters were subcloned upstream of the GUS reporter in the pCAMBIA1391Z vector. The CAMTA6 coding sequence was cloned downstream of the constitutive CaMV35S promoter (35S:CAMTA6) for overexpression (CAMTA6-OE). Each GUS-fused promoter vector, with or without CAMTA6-OE as indicated, was co-infiltrated into *N. benthamiana* leaves via Agrobacterium, followed by GUS staining of leaf discs from the infiltration region. Empty pCAMBIA1391Z vector served as control. The procedure was performed at least 10 times, and three representative replicates are shown. (b) RT-qPCR quantification of CAMTA6 transcript levels in CAMTA6-OE leaf discs.(c–e) Spectrophotometric GUS assay. Relative GUS activity was quantified using PNPG as a substrate (Jefferson, 1987) and normalized to the control (set as 1). Bold lines in each box represent the means ± SE (three biological experiments), and whiskers indicate ± SE. Top and bottom sides of the boxes correspond to the third and first quartiles, respectively. Asterisks indicate significant differences compared to the control by Student’s t-test (*P* < 0.005). Individual data points for each replicate are shown as dots overlaid on the box plots.

**
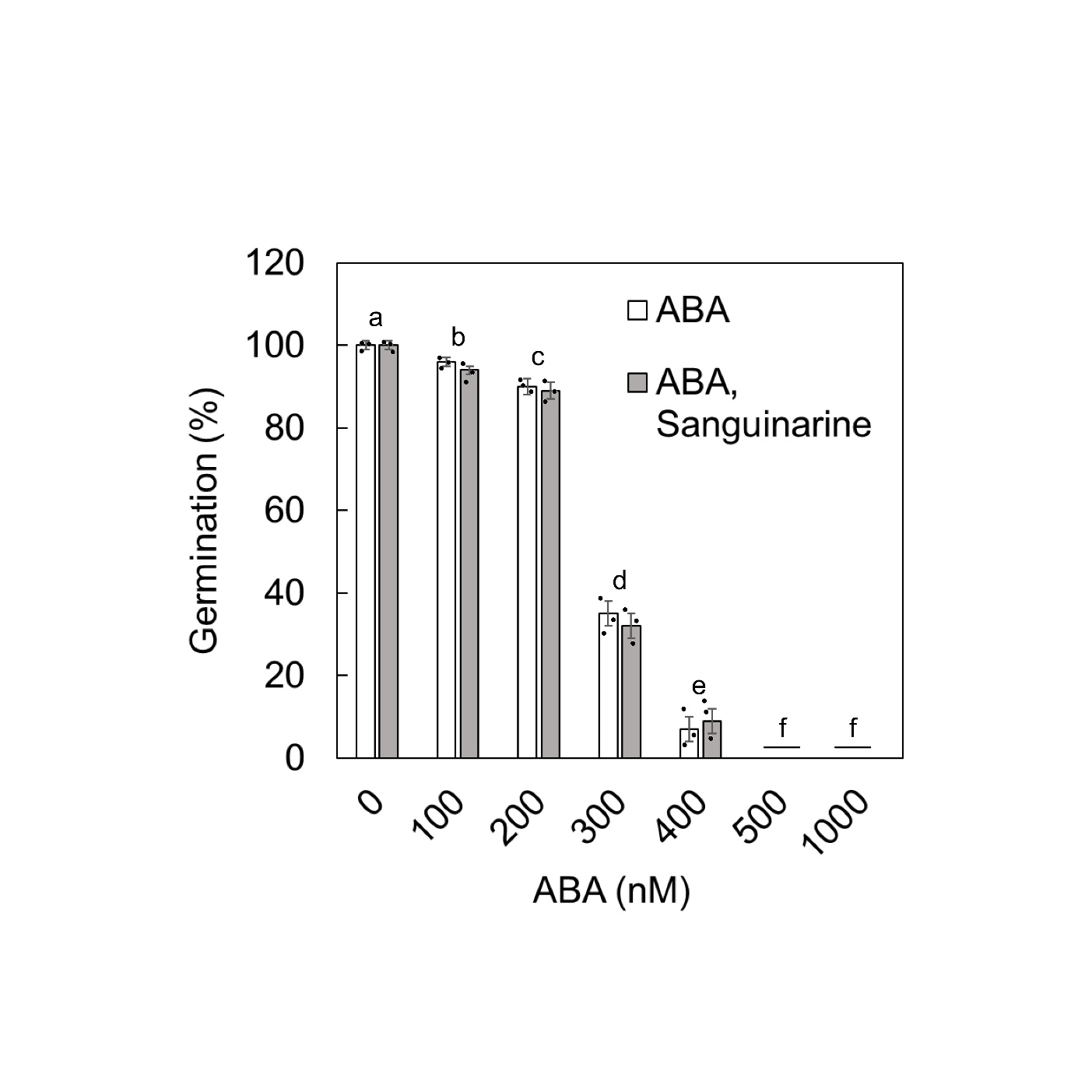
**

**Figure S4.** Sanguinarine does not affect ABA-mediated inhibition of Arabidopsis seed germination.

Wild type (Col-0) seeds were sown on agar-solidified 0.25X MS medium supplemented with ABA at the indicated concentrations, either alone or in combination with 1 µM sanguinarine. Germination rates were scored 5 days after plating. Data are presented as means ± SD from three independent biological replicates (~50 seeds per replicate). Different lowercase letters indicate significant differences (*P* < 0.001, Tukey’s HSD post hoc test). Individual data points for each replicate are shown as dots overlaid on the bars.

***
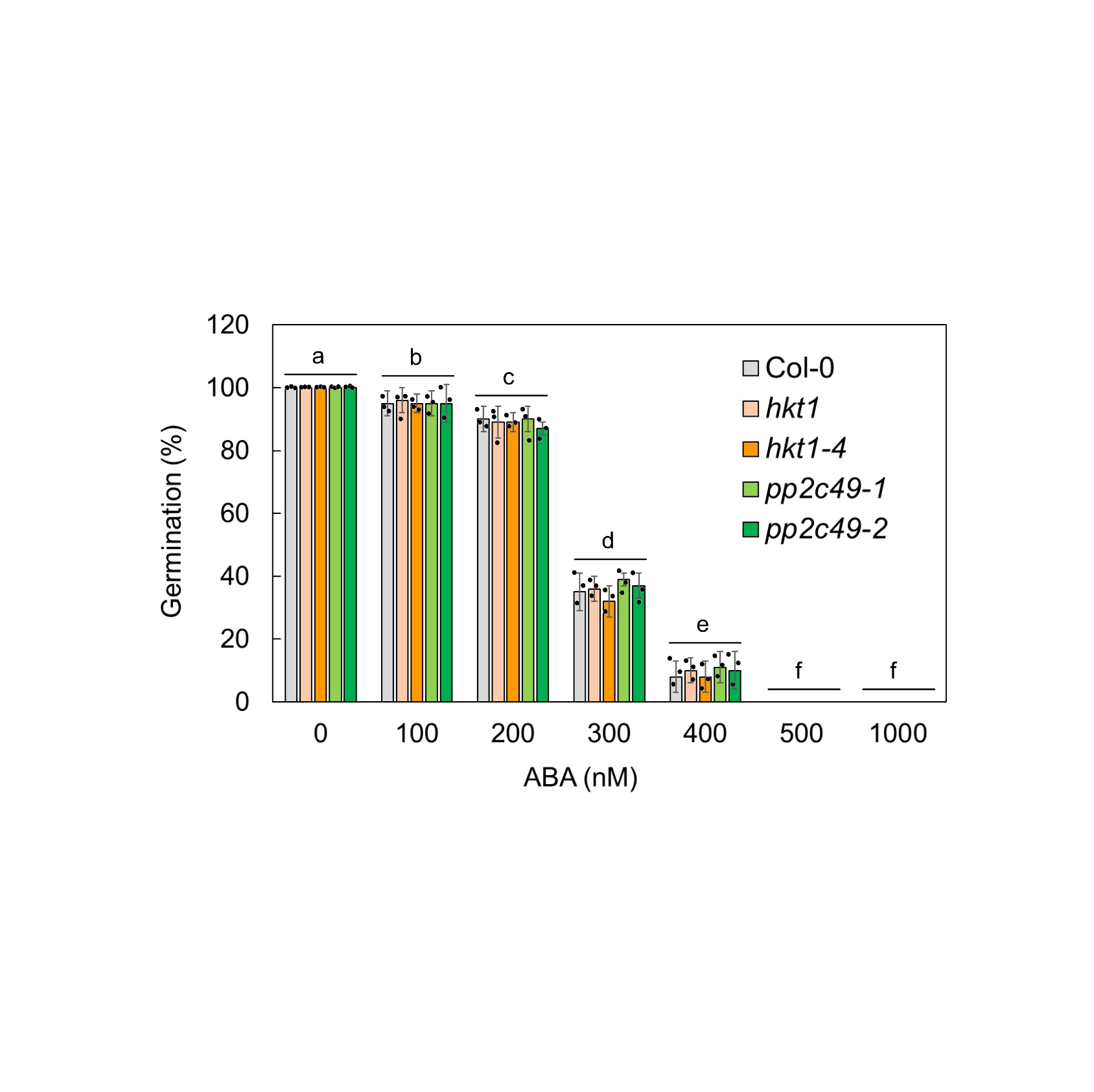
***

**Figure S5**. ABA inhibits germination in wild type and mutant Arabidopsis seeds.

Seeds of wild type (Col-0) and *hkt1*, *hkt1-4*, *pp2c49-1*, and *pp2c49-2* mutants were sown on agar-solidified 0.25X MS medium supplemented with ABA at the indicated concentrations. Germination was scored 5 days after plating. Data are presented as means ± SD from three independent biological replicates (~50 seeds per replicate). Different lowercase letters indicate significant differences (*P* < 0.01, Tukey’s HSD post hoc test). Individual data points for each replicate are shown as dots overlaid on the bars.


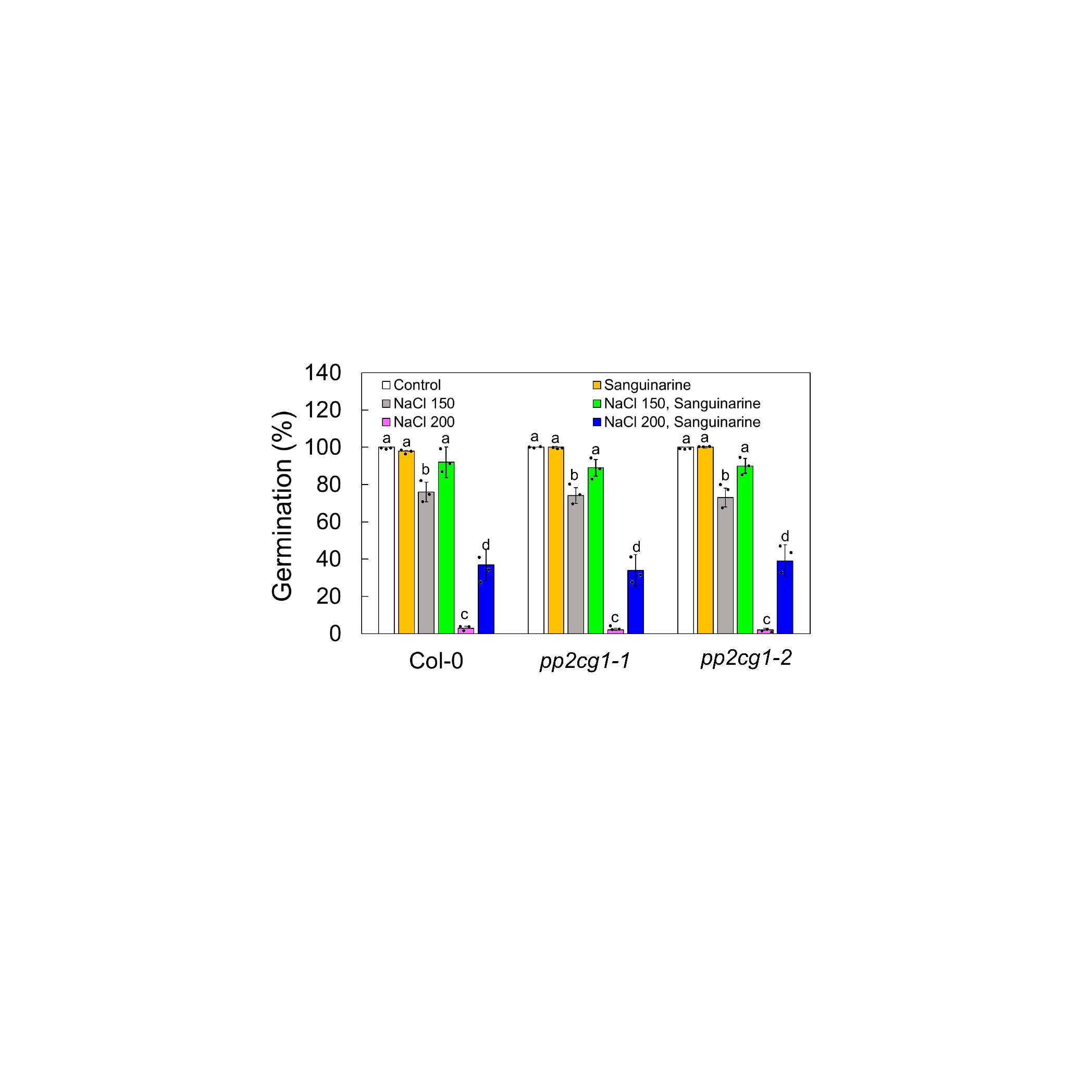


**Figure S6.** Effects of sanguinarine and NaCl on germination of wild type and *pp2cg1* mutant Arabidopsis seeds.

Seeds of wild type (Col-0) and *pp2cg1-1* and *pp2cg1-2* mutant alleles were sown on agar-solidified 0.25X MS medium under control conditions or supplemented with the indicated treatments: 1 µM sanguinarine (Sang.), 150 mM NaCl, 200 mM NaCl, Sang. + 150 mM NaCl, or Sang. + 200 mM NaCl. Germination rates were scored 5 days after plating. Data are presented as means ± SD from three independent biological replicates (~50 seeds per replicate). Different lowercase letters indicate significant differences by Tukey’s HSD post hoc test (*P* < 0.01). Individual data points for each replicate are shown as dots overlaid on the bars.


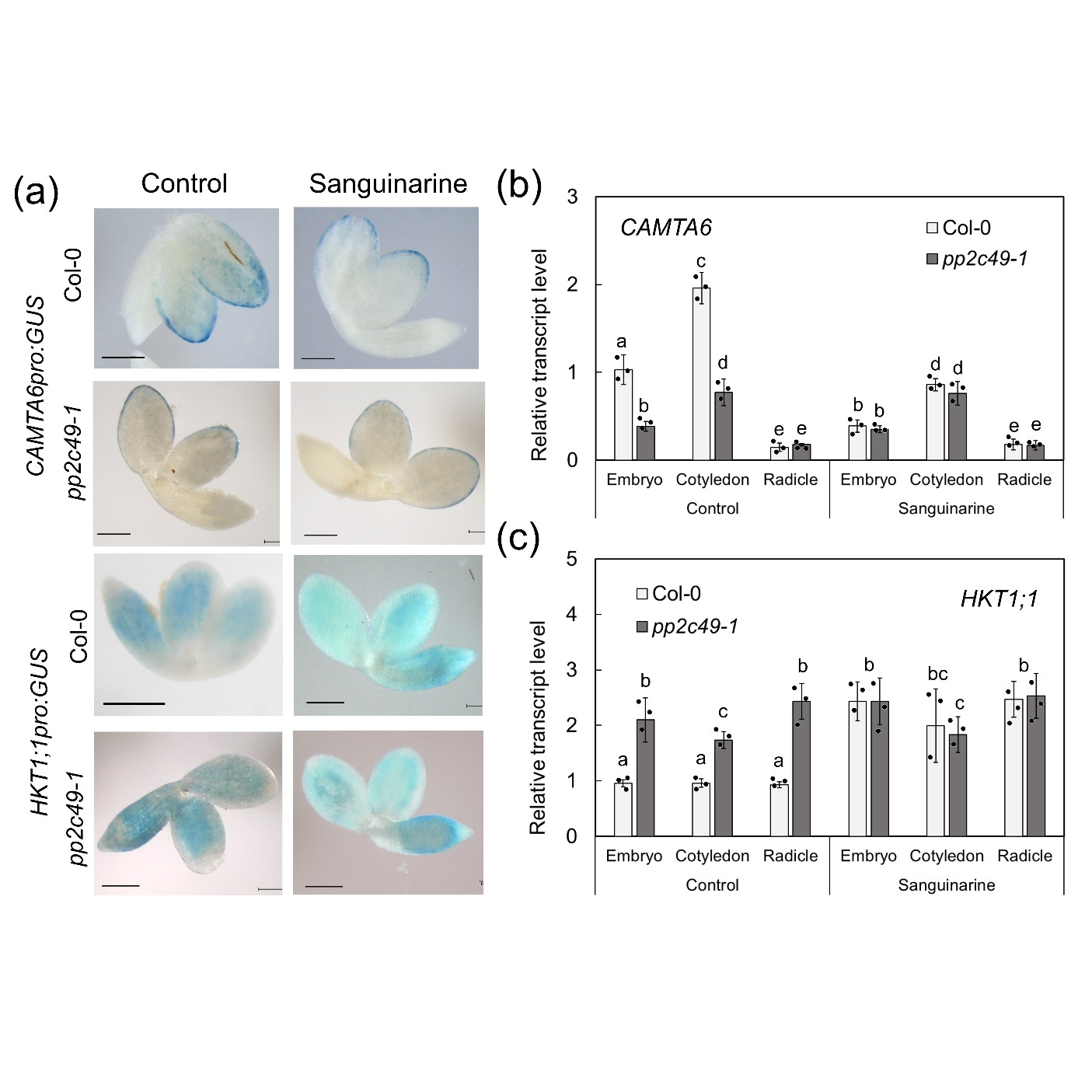


**Figure S7.** Sanguinarine (Sang.) modulates the expression of *CAMTA6* and *HKT1;1* in germinating Arabidopsis.

(a) GUS staining of germinating wild type (Col-0) or *pp2c49-1* embryos harboring the indicated constructs (*CAMTA6pro:GUS* and *HKT1;1pro:GUS*) treated with or without 1 µM sanguinarine, as indicated (bars = 0.5 mm). (b–c) RT-qPCR quantification of relative transcript levels of *CAMTA6* (b) and *HKT1;1* (c) in germinating embryos, cotyledons, and radicles of Col-0 and *pp2c49-1* under control or 1 µM sanguinarine treatments. Data are means ± SD (three biological experiments, ~30 seeds each) and different lowercase letters indicate significantly different values by Tukey’s HSD post hoc test (*P* < 0.01). Individual data points overlaid on the bars represent the mean value of technical triplicates for each biological replicate.

***
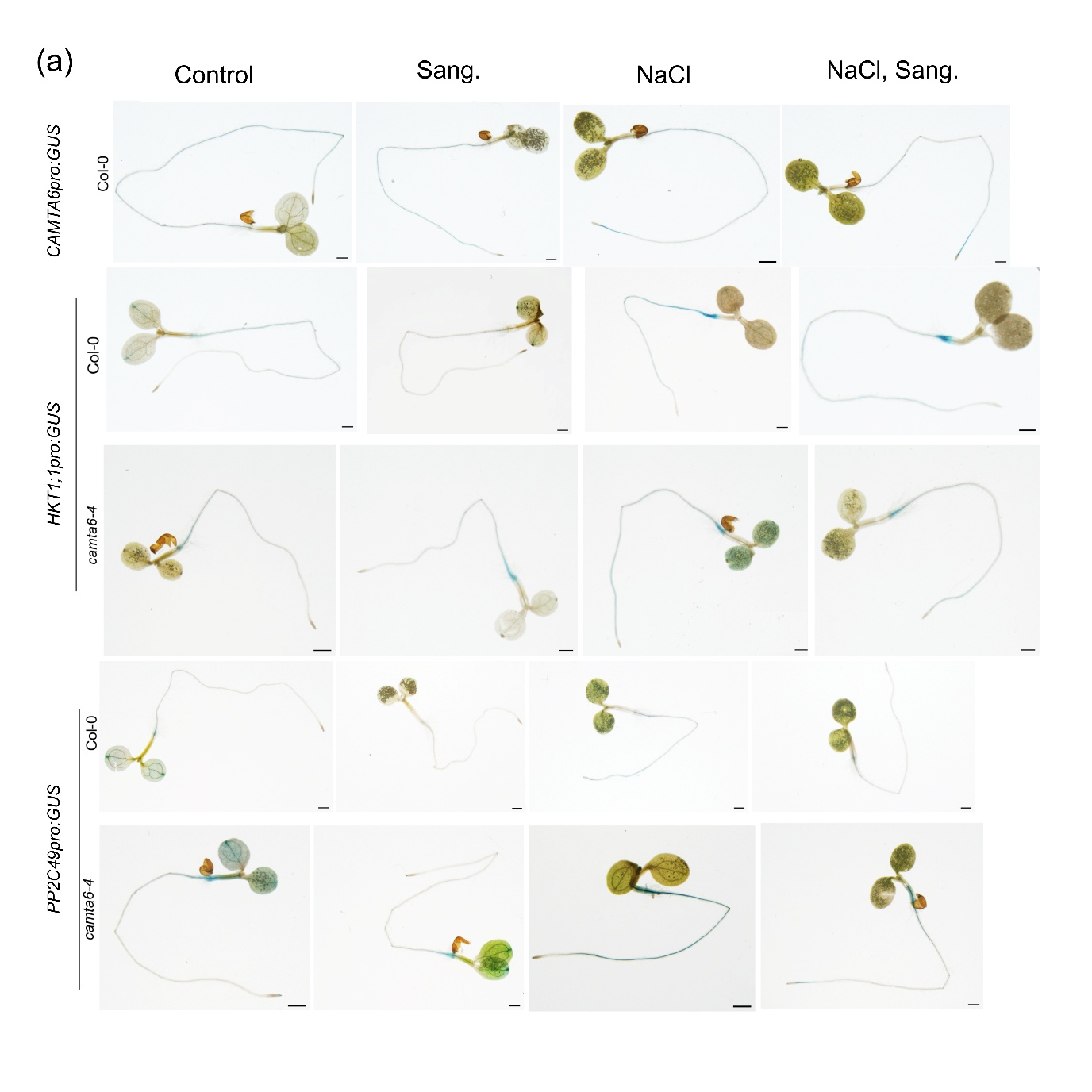
***

**
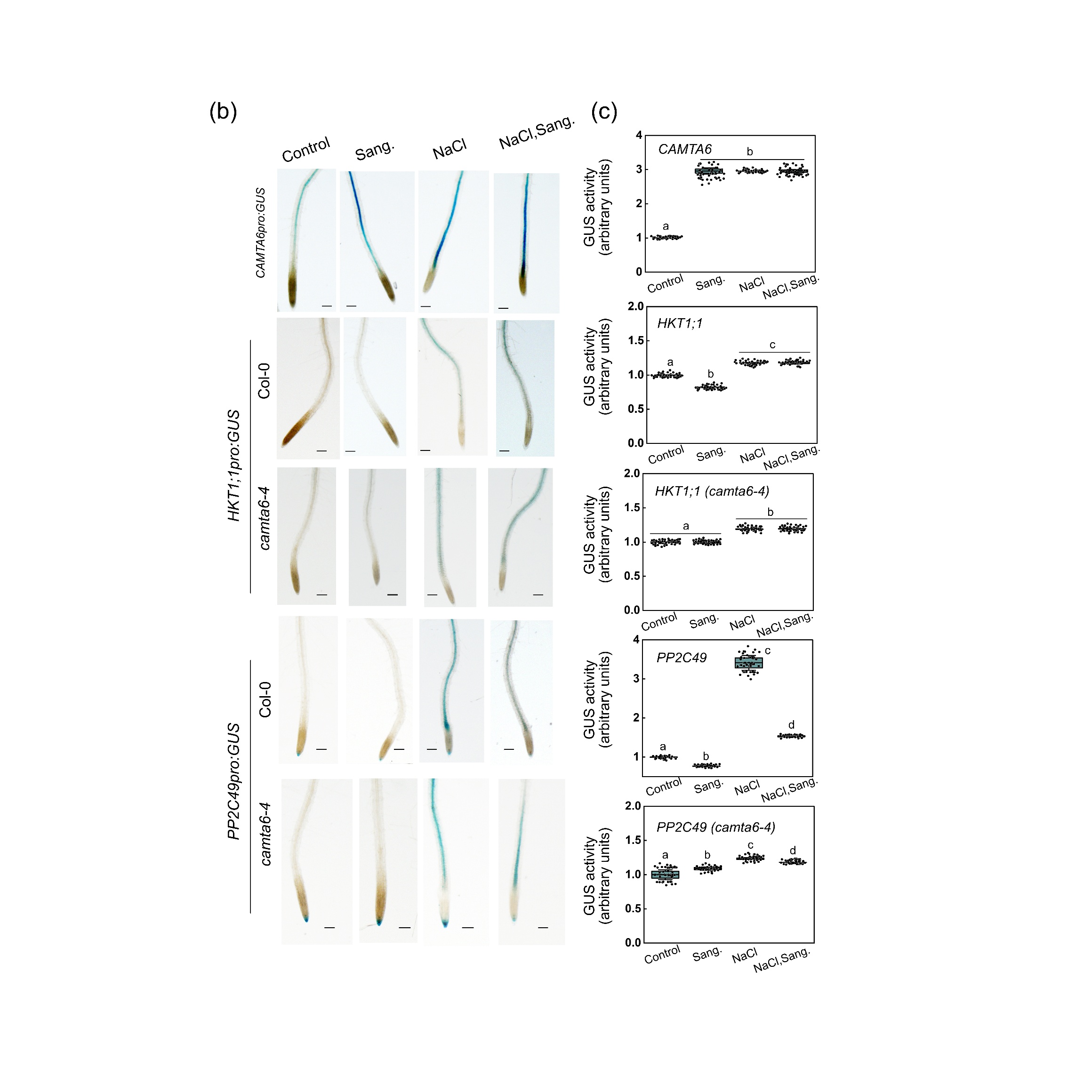
**

**Figure S8**. *CAMTA6*, *HKT1;1*, and *PP2C49* promoter activity in Arabidopsis seedlings treated with NaCl and/or sanguinarine (Sang.).

(a, b) Five-day-old seedlings of the indicated genotypes were treated with the specified chemicals (NaCl, 150 mM; Sang., 1 µM), GUS-stained (see Materials and Methods), and imaged using a stereomicroscope system. Whole seedlings are shown in (a) and young primary root zones in (b) (bars = 250 µm for a; 100 µm for b).

(c) Spectrophotometric GUS assay. Relative GUS activity of whole root tissue extracts was quantified using PNPG as a substrate (Jefferson, 1987). Bold lines in each box indicate the mean, and whiskers represent ± SD values (three independent biological experiments, n = 90). Top and bottom sides of the boxes correspond to the third and first quartiles. Different lowercase letters indicate significantly different values by Tukey’s HSD post hoc test (*P* < 0.005). Individual data points for each replicate are shown as dots overlaid on the box plots.

**
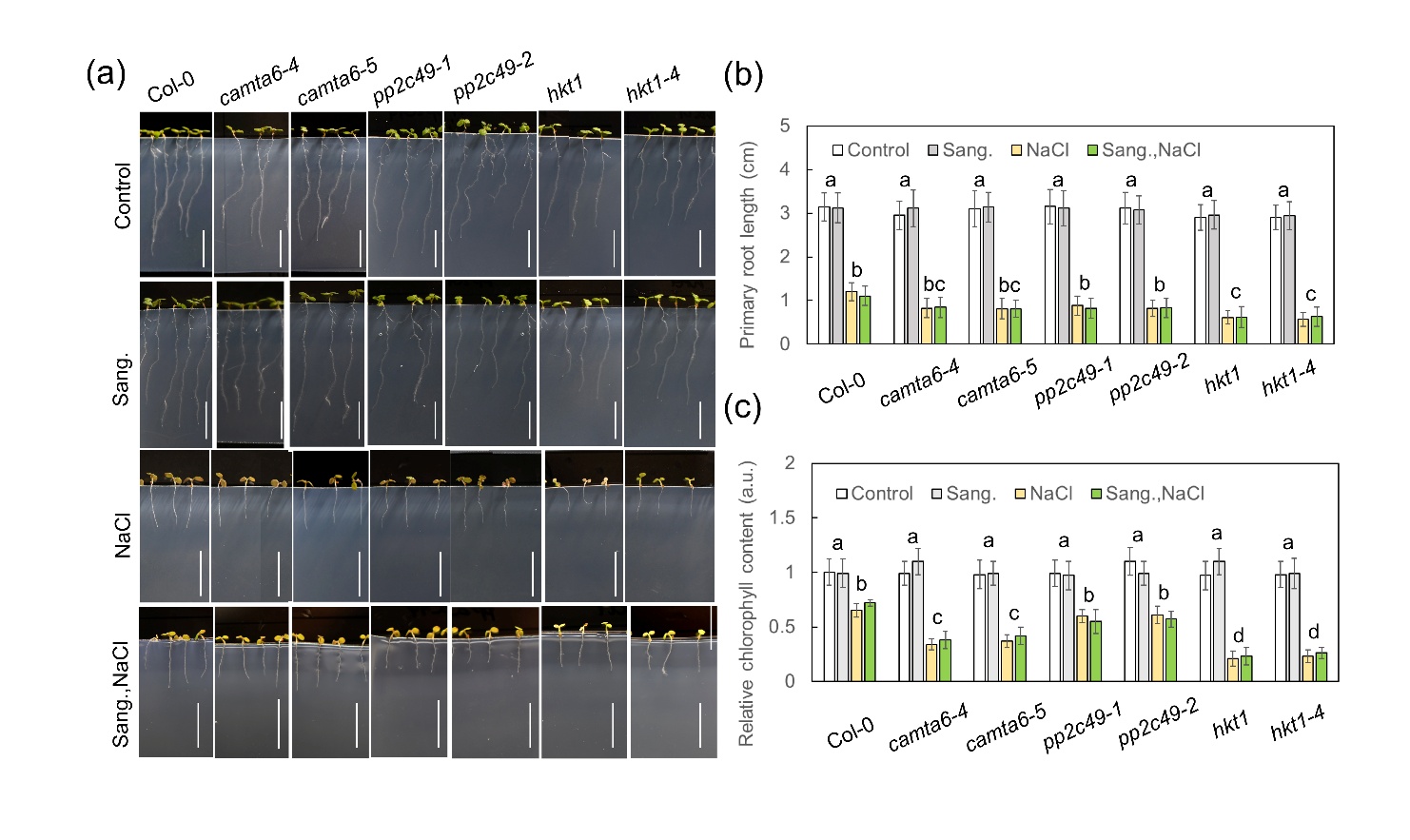
**

**Figure S9.** Effect of salt stress and sanguinarine (Sang.) on seedling growth and chlorophyll content of Arabidopsis genotypes.

(a) Representative images of seedlings of the indicated genotypes, including wild type (Col-0). Three-day-old seedlings were transferred to 0.25X MS medium supplemented with NaCl (150 mM), Sang. (1 µM), or a combination of both, and grown for an additional 4 days. Scale bars = 1 cm. (b) Primary root length of seedlings from the treatments described in (a). (c) Relative chlorophyll content of seedlings from the treatments described in (a). Data are presented as means ± SD (three independent biological experiments, n = 120). Different lowercase letters indicate significantly different values by Tukey’s HSD post hoc test (*P* < 0.001).


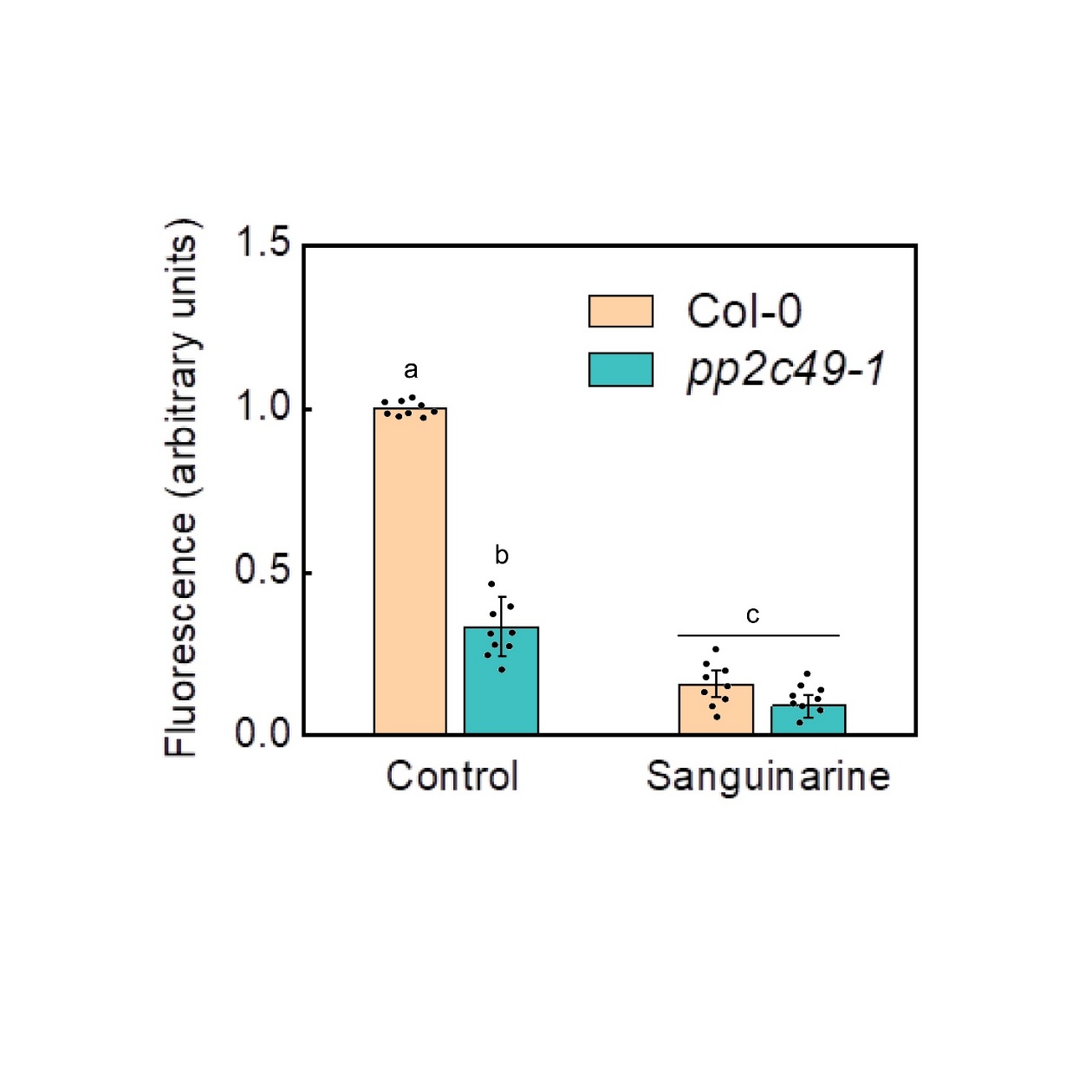


**Figure S10.** Phosphatase activity in roots of wild type and *pp2c49-1* Arabidopsis seedlings in the presence or absence of sanguinarine.

Crude protein extracts were prepared from roots of 4-day-old wild type (Col-0) and *pp2c49-1* seedlings. Phosphatase activity was measured using the fluorogenic substrate pNPP. Reactions contained 10 µg of total protein and were incubated with 10 mM pNPP at 30 °C for 30 min, with or without 1 µM sanguinarine. The release of p-nitrophenol was quantified by absorbance at 405 nm. Data are presented as means ± SD (three independent biological replicates, n=9). Activity values were normalized to the Col-0 control (set to 1.0). Different lowercase letters indicate significant differences according to Tukey’s HSD post hoc test (*P* < 0.01) following one-way ANOVA. Individual data points for each replicate are shown as dots overlaid on the bars.


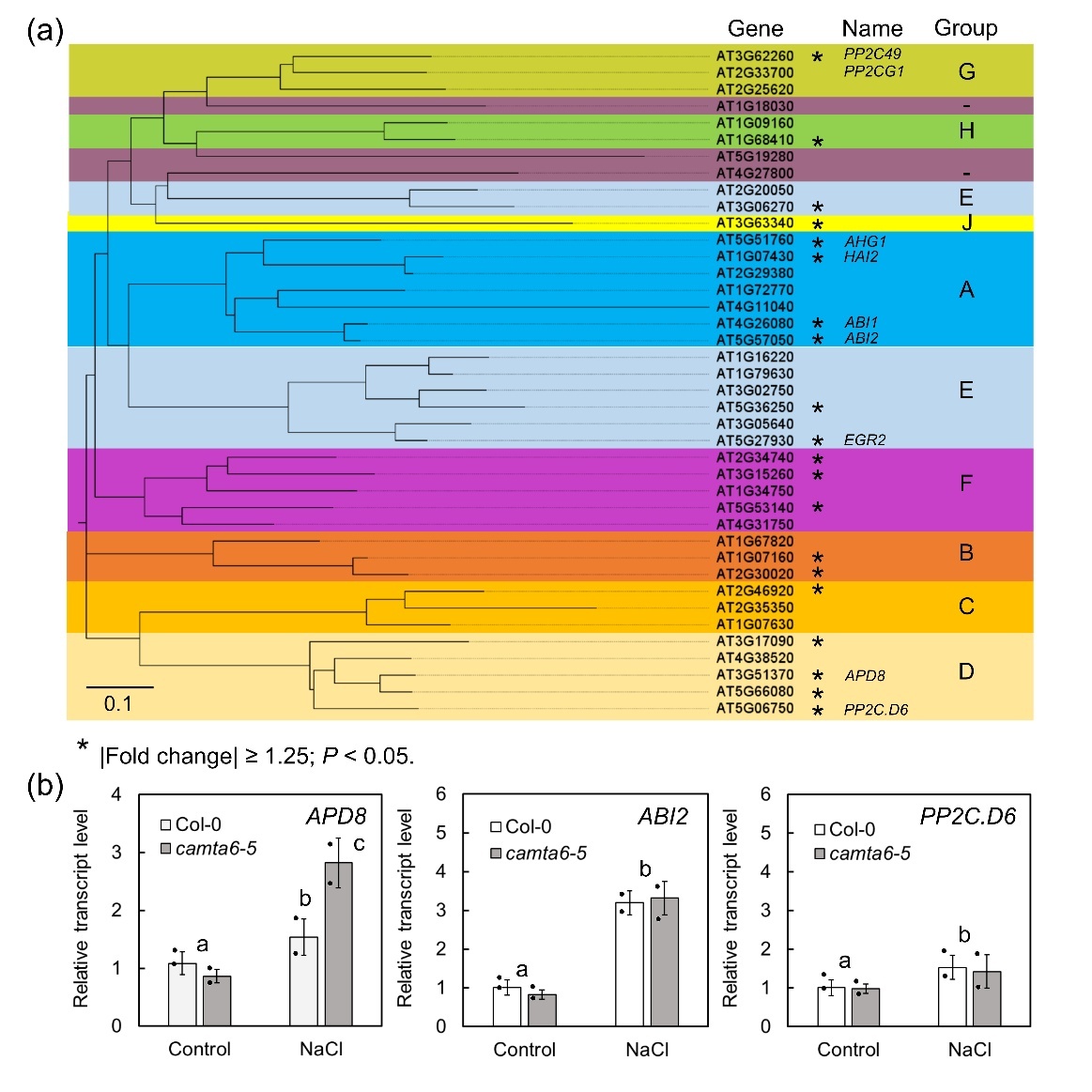


**Figure S11.** Phylogenetic classification and RT-qPCR validation of *PP2C* genes identified in transcriptome datasets.

(a) Phylogenetic classification of 40 overlapping PP2C genes. Genes were grouped into A–H and J based on maximum-likelihood phylogenetic analysis using RAxML and visualized with FigTree. Twelve genes meeting the selection criteria of absolute fold change ≥ 1.25 and *P* < 0.05 in at least one condition are indicated with an asterisk (*). Gene names and statistical values are provided in Supplementary Tables S1–S3. Scale bar represents 0.1 substitutions per site.

(b) RT-qPCR validation of representative *PP2C* genes in germinating seedlings. Relative transcript levels of *APD8*, *ABI2*, and *PP2C.D6* were determined in Col-0 and *camta6-5* germinating seedlings grown under control and NaCl stress conditions. Data represent the mean of two biological replicates ± SD (~30 seeds each). Distinct letters indicate statistically significant differences between groups (*P* < 0.05, Tukey’s HSD test). Individual data points overlaid on the bars represent the mean value of technical triplicates for each biological replicate. The RT-qPCR results confirm the expression trends observed in the RNA-seq analysis, supporting the role of CAMTA6 in early salt-stress responses.


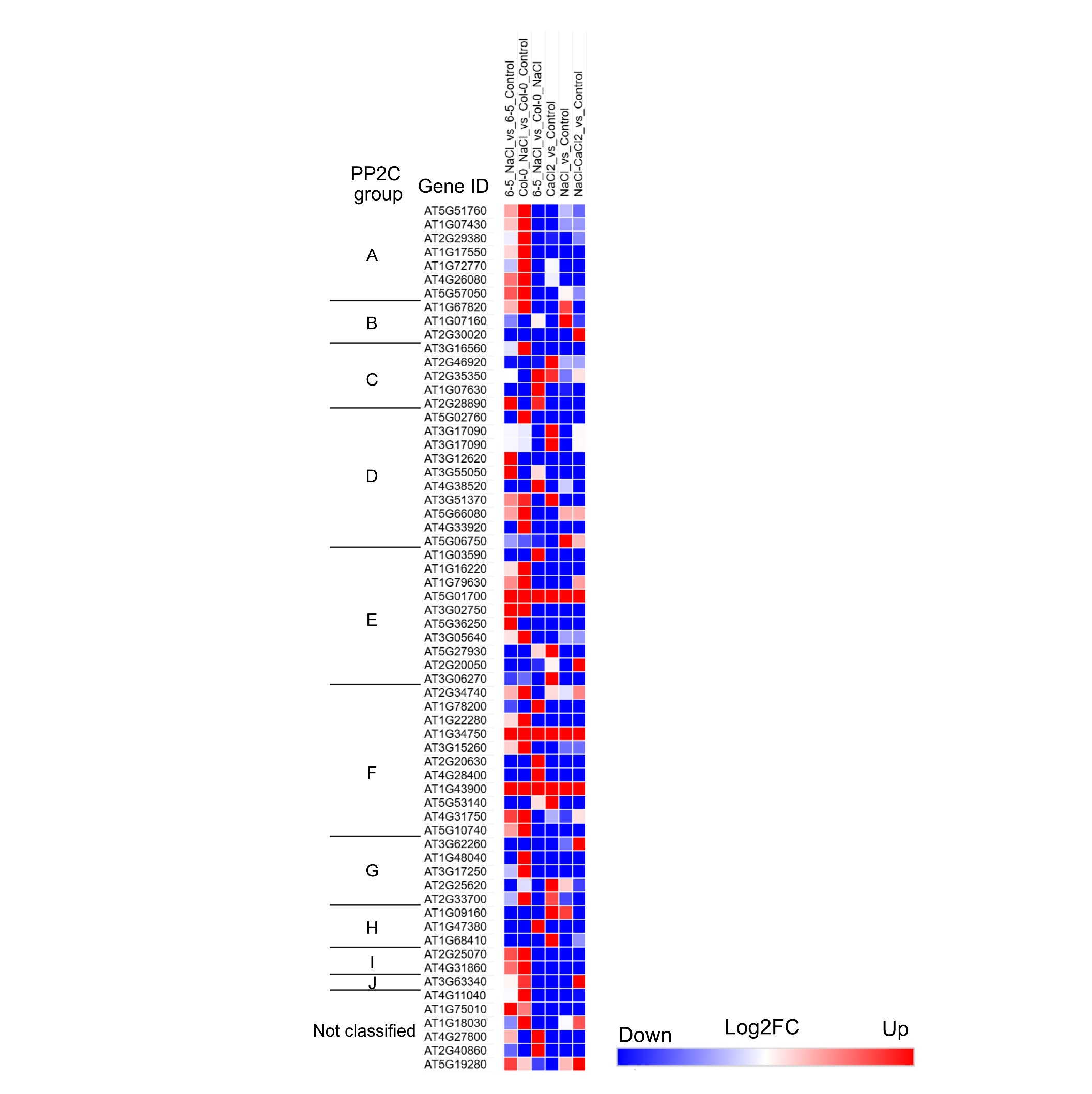


**Figure S12.** Distinct transcriptional profiles of PP2C group genes in wild type and Mutant Arabidopsis seedlings under salt stress.

Heatmap analysis displaying the transcriptional responses of PP2C group genes to salt stress in wild type (Col-0) and *camta6-5* mutant (6-5) seedlings, as visualized using the Morpheus web-based platform (Broad Institute). The phylogenetic classification of PP2C genes into groups A–H, I, and J is indicated on the left. Data represent the log_2_ fold change (Log2FC) of gene expression across different experimental comparisons (indicated at the top), including 6-5_NaCl vs 6-5_Control, Col-0_NaCl vs Col-0_Control, and mutant vs WT comparisons. Genes meeting the selection criteria of absolute fold change ≥ 1.25 and *P* < 0.05 in at least one condition are highlighted. The color scale indicates relative expression levels, with red representing Upregulation and blue representing Downregulation. Scale bar shows Log2FC levels. Analysis and visualization were performed using Morpheus (<https://software.broadinstitute.org/morpheus/>).

**
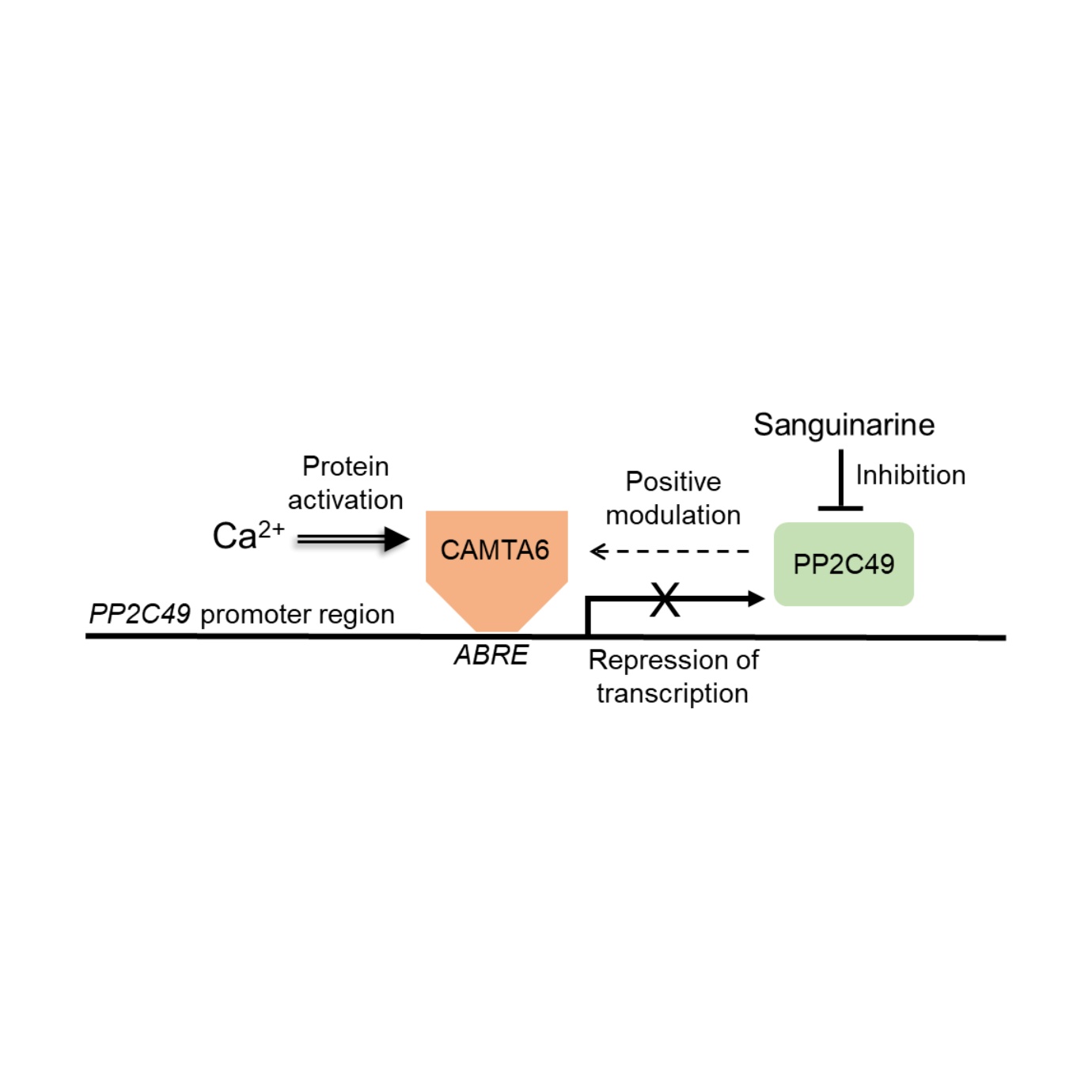
**

**Figure S13.** A proposed mechanistic framework for the CAMTA6–PP2C49 regulatory circuit and its spatial dynamics.

The proposed model focuses on the reciprocal molecular interactions within the CAMTA6–PP2C49 circuit across all embryonic tissues. Under basal conditions, CAMTA6 binds to the ABRE-box within the PP2C49 promoter to repress its transcription (Figure S3). Within this framework, Ca²⁺ signaling acts as a negative regulator of PP2C49 expression (Figure 2), likely by promoting CAMTA6-mediated repression to maintain homeostatic balance. This circuit is further characterized by a molecular feedback loop where PP2C49 exerts a positive modulation on CAMTA6 expression, as evidenced by the significant reduction in *CAMTA6* transcript levels in the *pp2c49-1* mutant (Figure S7). Consequently, the inhibition of PP2C49 activity by sanguinarine (Figure S10) disrupts this integrated circuit, leading to reduced CAMTA6 levels and the subsequent derepression of HKT1;1 (Figure 4). We propose that the uncontrolled, global upregulation of *PP2C49* observed in the *camta6-5* mutant results from the collapse of this regulation across all tissues. While the reciprocal influence of PP2C49 on CAMTA6 is supported by our findings, the precise molecular mechanism underlying this positive modulation warrants further investigation.

**Supporting table legends**

**Table S1.** List of differentially expressed *PP2C* genes identified in comparisons between wild type *Arabidopsis* (Col-0) and the *camta6-5* mutant under control and NaCl-treated conditions, in germinating seedlings. Genes that met the selection criteria of absolute fold change ≥ 1.25 and *P* < 0.05 were considered differentially expressed. The following pairwise comparisons are shown: (1) *camta6-5* NaCl vs. *camta6-5* control; (2) Col-0 NaCl vs. Col-0 control; (3) *camta6-5* control vs. Col-0 control; and (4) *camta6-5* NaCl vs. Col-0 NaCl. Data were extracted from Shkolnik et al. (2019).

**Table S2.** List of differentially expressed *PP2C* genes upregulated and downregulated in response to CaCl₂, NaCl, and their combination, compared to untreated controls, in germinating seedlings of wild type *Arabidopsis* (Col-0). Genes that met the selection criteria of absolute fold change ≥ 1.25 and *P* < 0.05 were considered differentially expressed. The following pairwise comparisons are shown: (1) CaCl₂ vs. control; (2) NaCl vs. control; (3) NaCl–CaCl₂ vs. control; (4) NaCl vs. CaCl₂; (5) NaCl–CaCl₂ vs. CaCl₂; and (6) NaCl–CaCl₂ vs. NaCl. Data were extracted from Chandran et al. (2023).

**Table S3.** List of differentially expressed *PP2C* genes shared between two independent datasets (Shkolnik et al., 2019; Chandran et al., 2023). Genes were included if they met the selection criteria of absolute fold change ≥ 1.25 and *P* < 0.05 in at least one comparison within each dataset. The table presents *PP2C* genes differentially expressed in germinating seedlings of wild type *Arabidopsis* (Col-0) in response to control, NaCl, CaCl₂, and combined NaCl-CaCl₂ treatments, and in the *camta6-5* mutant under control and NaCl-treated conditions.

**Table S4.** List of primers used in this study.

**Reference**

Jefferson, R. A. (1987). Assaying chimeric genes in plants: the GUS gene fusion system. *Plant Molecular Biology Reporter*, *5*, 387–405.
